# Modeling the association between *Aedes aegypti* ovitrap egg counts, multi-scale remotely sensed environmental data and arboviral cases at Puntarenas, Costa Rica (2017–2018)

**DOI:** 10.1016/j.crpvbd.2021.100014

**Published:** 2021-02-09

**Authors:** Luis Fernando Chaves, José Angel Valerín Cordero, Gabriela Delgado, Carlos Aguilar-Avendaño, Ezequías Maynes, José Manuel Gutiérrez Alvarado, Melissa Ramírez Rojas, Luis Mario Romero, Rodrigo Marín Rodríguez

**Affiliations:** a Vigilancia de la Salud, Ministerio de Salud, San José, San José, Apartado Postal 10123-1000, Costa Rica; b Coordinación Regional, Programa Nacional de Manejo Integrado de Vectores, Región Pacífico Central, Ministerio de Salud, Puntarenas, Puntarenas, Código Postal 60101, Costa Rica; c Oficina Central de Enlace, Programa Nacional de Manejo Integrado de Vectores, Ministerio de Salud, San José, San José, Apartado Postal 10123-1000, Costa Rica; d Departamento de Patología, Escuela de Medicina Veterinaria, Universidad Nacional, Heredia, Heredia, Apartado Postal 304-3000, Costa Rica

**Keywords:** Oviposition, MODIS, Landsat 8, Sentinel 2, Schmalhausen’s law, Model selection, Synchrony, Syndemic arboviruses

## Abstract

Problems with vector surveillance are a major barrier for the effective control of vector-borne disease transmission through Latin America. Here, we present results from a 80-week longitudinal study where *Aedes aegypti* (L.) (Diptera: Culicidae) ovitraps were monitored weekly at 92 locations in Puntarenas, a coastal city in Costa Rica with syndemic Zika, chikungunya and dengue transmission. We used separate models to investigate the association of either *Ae. aegypti*-borne arboviral cases or *Ae. aegypti* egg counts with remotely sensed environmental variables. We also evaluated whether *Ae. aegypti*-borne arboviral cases were associated with *Ae. aegypti* egg counts. Using cross-correlation and time series modeling, we found that arboviral cases were not significantly associated with *Ae. aegypti* egg counts. Through model selection we found that cases had a non-linear response to multi-scale (1-km and 30-m resolution) measurements of temperature standard deviation (SD) with a lag of up to 4 weeks, while simultaneously increasing with finely-grained NDVI (30-m resolution). Meanwhile, median ovitrap *Ae. aegypti* egg counts increased, and respectively decreased, with temperature SD (1-km resolution) and EVI (30-m resolution) with a lag of 6 weeks. A synchrony analysis showed that egg counts had a travelling wave pattern, with synchrony showing cyclic changes with distance, a pattern not observed in remotely sensed data with 30-m and 10-m resolution. Spatially, using generalized additive models, we found that eggs were more abundant at locations with higher temperatures and where EVI was leptokurtic during the study period. Our results suggest that, in Puntarenas, remotely sensed environmental variables are associated with both *Ae. aegypti*-borne arbovirus transmission and *Ae. aegypti* egg counts from ovitraps.

## Highlights

• We sampled *Ae. aegypti* eggs using ovitraps for 80 weeks in Puntarenas, Costa Rica.
• We were able to correlate *Ae. aegypti* egg-counts and arboviral cases with remotely sensed data.
• Egg counts and arboviral cases were correlated with temperature and vegetation indices.
• Arboviral cases were not associated with egg counts.

## Graphical abstract

**Figure:**
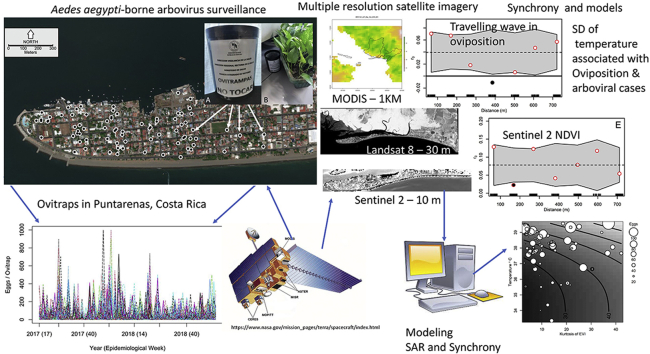

## 1 Introduction

Pathogens transmitted by *Aedes aegypti* (L.) (Diptera: Culicidae) are a major health problem throughout Latin America and the Caribbean (Wallace et al., 2018). Dengue (San Martín et al., 2010; Perez et al., 2019), Zika (Colón-González et al., 2017) and chikungunya (Yactayo et al., 2016) have become syndemic through most of the region, and problems with vector surveillance and control seem to be a major driver for the impact of these diseases through Latin America (Fernández-Salas et al., 2015; Weaver et al., 2016) and elsewhere (Weaver & Reisen, 2010; Weaver et al., 2018). One tool that has proved highly efficient for the surveillance of *Ae. aegypti* is the systematic ovitrap sampling, which can be used for entomological surveillance (CENAPRECE, 2015), both to detect its presence (Baak-Baak et al., 2016; Ortega-Morales et al., 2018) and estimate its abundance (Tovar-Zamora et al., 2019). Ovitraps can also be used for mosquito population control (Ritchie et al., 2003, 2004; Barrera et al., 2014, 2017; Martin et al., 2019).

In Costa Rica, dengue, chikungunya and Zika are the most common arboviruses affecting humans, and account for the highest vector-borne disease burden in the country, given their high case number, which is in the magnitude of thousands of annual cases (Soto-Garita et al., 2016; Vigilancia de la Salud, 2019). This case load is well above what is observed for other vector-borne diseases, such as cutaneous leishmaniasis, where annual cases amount to a few hundred (Chaves et al., 2008), or malaria, which is on the brink of elimination (Chaves et al., 2020a). In Costa Rica, dengue transmission has been traditionally located at low altitudes, on coastal regions from the Pacific and Caribbean basins (Mena et al., 2011). On the Pacific basin, the county of Puntarenas has experienced high transmission of *Ae. aegypti*-borne pathogens over recent decades. For example, a serological survey found over 90% of the people exposed to dengue virus (Lee-Lui et al., 2008). Moreover, Puntarenas county is regularly among the top 10% of counties reporting dengue cases in Costa Rica (Vigilancia de la Salud, 2019).

*Aedes aegypti* has a long history in Puntarenas and was documented to be present by 1920 (Alfaro, 1921; Serre, 1921) and the late part of the 1930s (Kumm et al., 1940). In 1961, the country was certified as free from *Ae. aegypti* as part of yellow fever eradication efforts (Soper, 1967), a status held until at least 1988 (Gubler, 1989). However, in 1993 local dengue transmission started, implying that *Ae. aegypti* was again present in Puntarenas (Sáenz et al., 1999). Recent studies have identified that key larval habitats of *Ae. aegypti* in Puntarenas include artificial discarded containers, but also water holding tanks, and other containers with active domestic use (Troyo et al., 2009; Marín Rodríguez & Díaz Ríos, 2012). A couple of studies have described insecticide resistance patterns in *Ae. aegypti* from Puntarenas. Bisset et al. (2013) reported resistance to the pyrethroid deltamethrin and to the organophosphate temephos used for larval control. Zardkoohi et al. (2020) confirmed the resistance to deltamethrin, and the emergence of resistance to the pyrethroid cypermethrin, linking pyrethroid resistance to the co-occurrence of the V1016I and F1534C *kdr* mutations in the voltage-gated sodium channel gene.

Several studies have linked weather changes with both *Ae. aegypti* abundance and arboviral pathogen transmission (Scott et al., 2000; Johansson et al., 2009; Chaves, 2017a; Reinhold et al., 2018). However, no attempt has been made at linking temporal changes in *Ae. aegypti*-borne arboviral transmission, and *Ae. aegypti* abundance, with weather variables at Puntarenas, which is, and has historically been, a major focus for dengue transmission in Costa Rica. This potential association is particularly important because a global meta-analysis found limited evidence for dengue transmission prediction based on data from entomological surveys of *Ae. aegypti* aquatic stages (Bowman et al., 2014). One major problem to test if dengue transmission was associated with entomological indicators was the lack of a spatially standardized sampling (Bowman et al., 2014), which is more feasible with ovitraps (CENAPRECE, 2015). In Costa Rica, ovitraps have been successfully used to survey mosquito biodiversity (Chaverri et al., 2018) and to study mosquito population dynamics (Romero et al., 2019). Moreover, the Costa Rican National programme for integrated vector management started to use ovitraps to guide vector control in 2017, as done elsewhere in Latin America (Regis et al., 2008; CENAPRECE, 2015; PAHO, 2017). All these conditions make Puntarenas an ideal site to test if arboviral cases can be associated with *Ae. aegypti* egg counts from ovitraps.

Here, we present the results of an 80-week longitudinal study where weekly observations on the number of eggs at 92 ovitrap locations were recorded between 2017 and 2018. We tested whether remotely sensed environmental data and *Ae. aegypti* egg counts from ovitraps can be associated with arboviral transmission in Puntarenas, a city on the Pacific Coast of Costa Rica. Additionally, we also tested whether egg counts were associated with remotely sensed environmental variables.

For our analysis, we employed satellite images with different resolution. Specifically, we included Sentinel 2 (10 m resolution), Landsat 8 (30 m) and MODIS (250 m and 1 km). We used several temporal, spatio-temporal and spatial modelling techniques. We started by (i) performing an ancillary analysis comparing land surface temperature estimates obtained with MODIS images for the Puntarenas city area, where cases were recorded, with estimates based on Landsat 8 images for the Puntarenas peninsula area, the subsection of Puntarenas city where most of the transmission is assumed to occur (Marín Rodríguez & Díaz Ríos, 2012).

We then developed (ii) time series models to assess the association between the remotely sensed environmental variables with *Ae. aegypti* egg counts and *Ae. aegypti*-borne arboviral cases. We specifically employed seasonal autoregressive (SAR) models given their ease for fitting and interpretation (Chaves & Pascual, 2007). Among other things, these models allow testing for the significance of environmental variables at time lags whose association with the studied time series is not an artifact of a similar, but unrelated, seasonality (Priestley, 1988). These models allow to evaluate if the arboviral cases were associated with egg counts and remotely sensed environmental variables at different time lags. This analytic framework was also used to assess the association between egg counts and remotely sensed environmental variables at different lags.

We also performed (iii) a synchrony analysis of *Ae. aegypti* egg counts and the remotely sensed environmental variables based on Landsat 8 and Sentinel 2 images. This is an innovative analysis to study spatio-temporal patterns in mosquito oviposition, which we have previously used to study mosquito larval (Chaves et al., 2020b) and adult (Chaves, 2017b) abundance patterns. Briefly, a synchrony analysis shows the degree of concerted fluctuation between values of a studied variable, egg counts in this study, as a function of the distance separating the sampling locations (Chaves et al., 2013). This analysis allows to test if the correlations between egg counts are synchronous, i.e. not changing with distance. Alternatively, the correlations can decay with distance as expected under dispersal limitation, or have a fluctuating sign, changing from positive to negative, with distance, as expected when dispersal occur in traveling waves (Ranta et al., 2006). Thus, the synchrony analysis provides a description of the type of spatial heterogeneity in oviposition, measured through time. The obtained oviposition synchrony can also be compared with the synchrony in environmental data to establish a dependence of the former on the latter (Chaves et al., 2013).

We finished by performing a (iv) spatial analysis of the mean number of *Ae. aegypti* eggs at each sampled location with average values for the remotely sensed environmental variables, based on Landsat 8 and Sentinel 2 images, at the pixels where traps were located. For this analysis, we employed generalized additive models. This modelling framework enables to easily incorporate nonlinearities in the association between variables (Faraway, 2006). This analysis allowed to test if average egg counts were associated with average environmental conditions estimated from finely-grained (30 m and 10 m) remotely sensed data.

## 2 Materials and methods

### 2.1 Study site

**Fig. 1:**
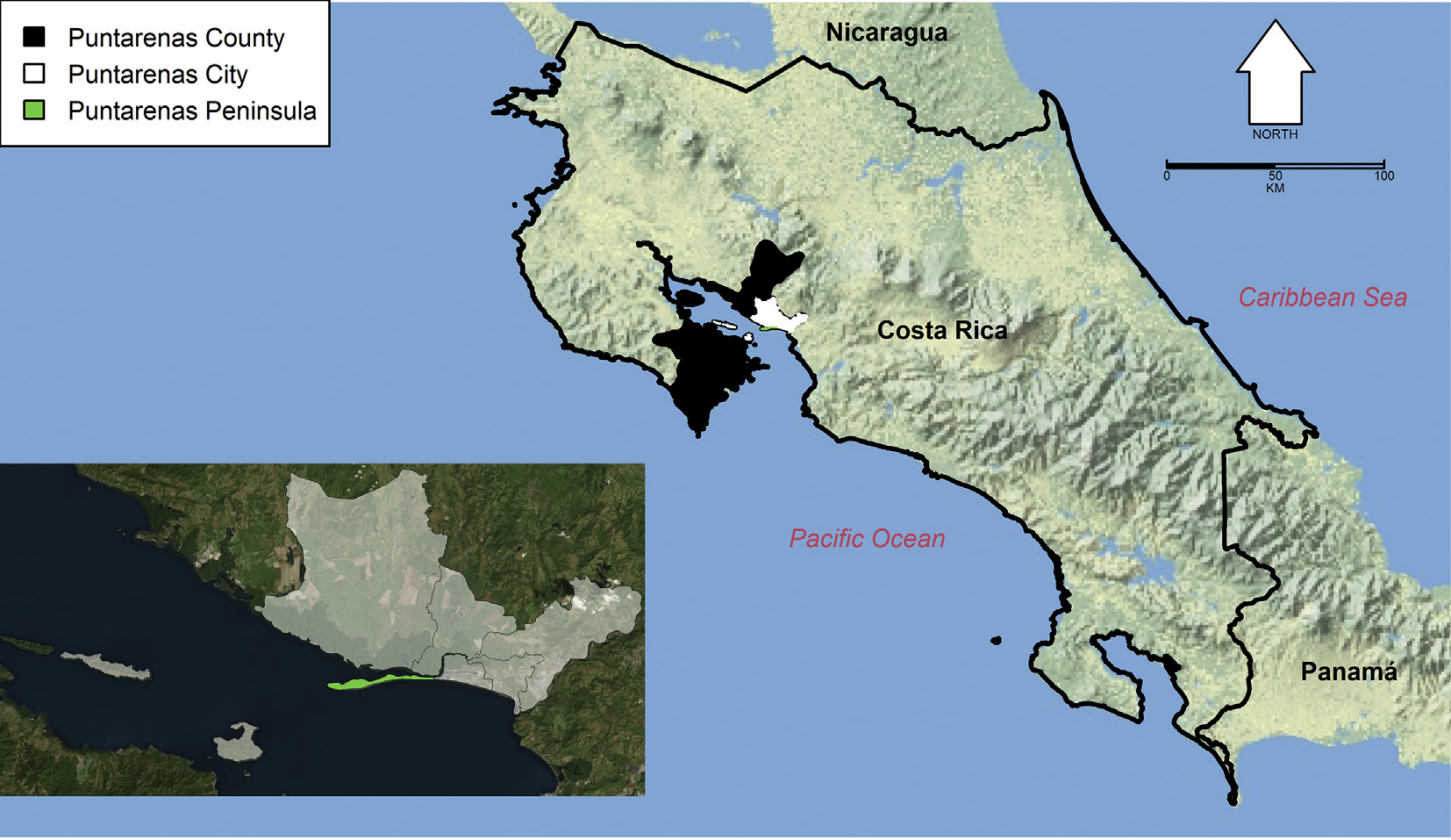
Map of Costa Rica and Puntarenas city. The map highlights the location of Puntarenas county and the inset map highlights the urban area of Puntarenas city and the Puntarenas peninsula part of Puntarenas district. The base image for the main map is from a public domain map from the US National Park Service (USNPS, 2020), while the inset base image is courtesy of Google maps

Our study was carried out in Puntarenas city (9°58′34.50″N, 84°50′18.10″W), the largest city on the Pacific coast of Costa Rica (Fig. 1), capital of Puntarenas county and Puntarenas province. The urban area of Puntarenas city is formed by Puntarenas, Chacarita, Barrancas, El Roble and Pitahaya districts (Fig. 1). The city has a population of 81,187 inhabitants, according to the last census (2011), and 193.98 km^2^ of area, for a population density of 418 inhabitants/km^2^ (INEC, 2012). Puntarenas peninsula, the peninsular part of Puntarenas district (Fig. 1), concentrates government, education and commercial activities of Puntarenas city (Villanueva, 2009). Bus and ferry terminals that serve as transportation hubs for Puntarenas city, Puntarenas county and the Pacific basin of Costa Rica are also located in the Puntarenas peninsula (Chen et al., 2017).

Puntarenas city has a marked seasonal climatic pattern with a dry season from December to April, and a rainy season over the rest of the year (World Meteorological Organization, 2020). The average annual total cumulative rainfall is 1,600 mm, while temperature has an annual average of 32.8 °C, which is about 2.1 °C hotter during the dry season than the rainy season (World Meteorological Organization, 2020).

### 2.2 Mosquito sampling

**Fig. 2:**
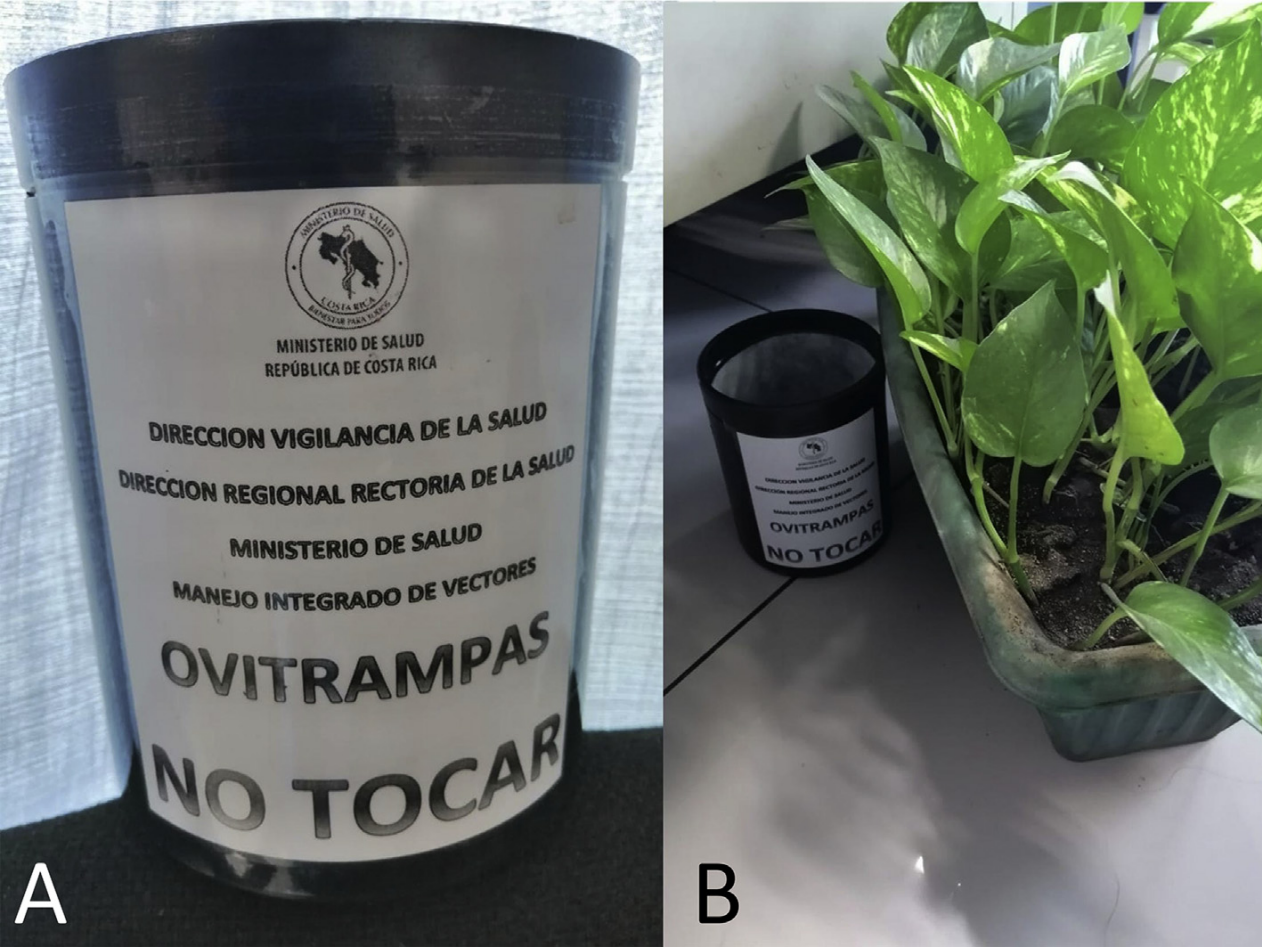
Ovitraps. **A** Ovitrap label indicating its use for dengue vector surveillance and providing contact information for the Programa de Manejo Integrado de Vectores of Costa Ricaʼs Ministry of Health. **B** Field deployment of an ovitrap, next to a plant pot, a common larval habitat for *Aedes aegypti* in Puntarenas, Costa Rica

Mosquitoes were sampled using ovitraps made with PVC plastic. The ovitraps have a cylindrical shape (diameter of 11 cm and height of 14 cm), with a closed and an open end (Fig. 2A) and were donated by the Mexican Government through the Center for Infectious Disease Control and Prevention (CENAPRECE), a branch of Mexico’s Secretary of Health. The ovitraps have two 5 mm in diameter openings 8 cm above the closed end, thus having a water holding volume of 750 ml. Ovitraps were set using Scott® paper towels (Kimberly Clark Co., Neenah, WI, USA), which were placed around the internal side of the ovitrap. Following the recommendations from CENAPRECE (2015) we filled the ovitraps with 750 ml of tap water. This water came from the city water distribution network, where chlorine concentrations are strictly monitored and fluctuate between 0.3 and 0.6 mg/l (Marín Mena, 2007). The paper towels were added to sample *Ae. aegypti* eggs, given the oviposition behavior of this species which places eggs on surfaces above the water line (Chadee & Corbet, 1987). Traps were labeled (Fig. 2A) and deployed (Fig. 2B) following a protocol developed by the CENAPRECE (2015).

**Fig. 3:**
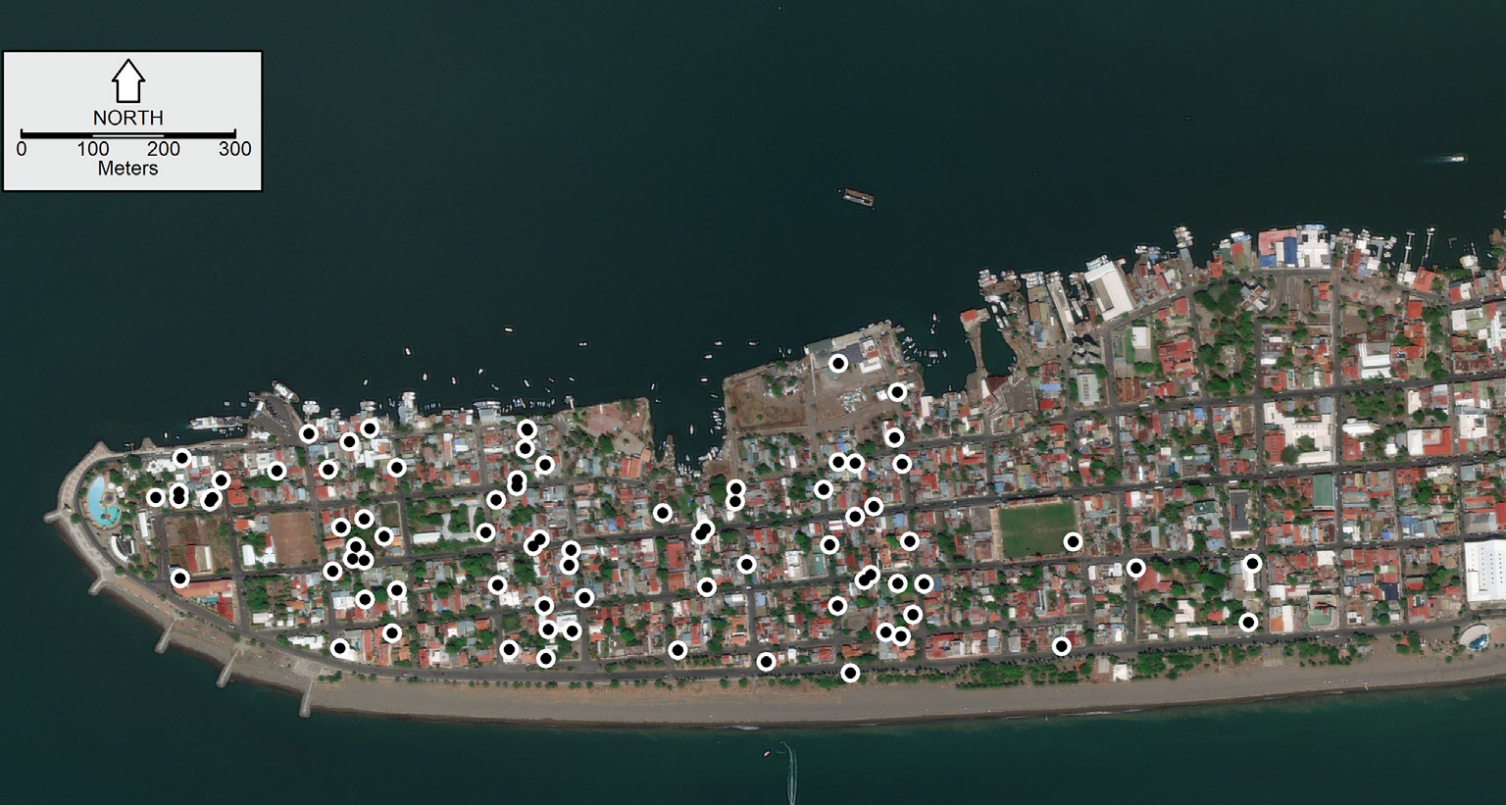
Ovitrap sampling locations. Ovitraps are indicated by dots on the map. The location of only 83 of 92 the ovitraps are shown since 9 records had ambiguous addresses that could not be found using Google maps. The base image is courtesy of Google maps

A total of 92 traps were deployed on April 17th, 2017 (epidemiological week 16) through the Puntarenas peninsula (Fig. 3). Both the water and the paper towels were replaced every Monday from epidemiological week 17 of 2017 (April 24th 2017) to epidemiological week 44 of 2018 (October 29th 2018). This was done to avoid biases in mosquito sampling related to ovitrap water age and/or conspecific presence (Chadee, 2009). Ovitraps were placed inside the houses (*n* = 90) or outside the houses but within the household space, e.g. the backyard, specifically an area outside the houses where a sink for washing clothes (“pila” in the Spanish of Costa Rica) is traditionally located (*n* = 2). Ovitraps were placed next to the walls defining the residential premises, i.e. within approximately within one meter from the wall (within 1 m), in places selected by the homeowners, but restricted to the first floor of multi-story buildings. Traps were deployed following CENAPRECE (2015) recommendations, i.e. a maximum of 4 ovitraps were deployed by residential block, “manzana” in Spanish, trying to ensure a minimal distance between any pair of traps was 20 m, something done because of the finely-grained nature of *Ae. aegypti* oviposition (Harrington et al., 2008). Homeowners were asked for consent to place the ovitraps in their households as part of efforts for arbovirus transmission surveillance.

In light of insights about dengue transmission in public spaces with a high turnover of visitants and human movement (Stoddard et al., 2009; Arunachalam et al., 2010; Smith et al., 2014), ovitraps were located within the Puntarenas peninsula. This decision was made given that Puntarenas peninsula serves as a hub for human movement within Puntarenas city (Chen et al., 2017) and because previous studies have shown persistent mosquito infestations in the area (Marín Rodríguez & Díaz Ríos, 2012) suggesting that the peninsula is a place where most *Ae. aegypti*-borne arboviruses are transmitted among inhabitants of the larger Puntarenas city (Marín Rodríguez & Díaz Ríos, 2012).

Ovitraps were georeferenced using the recorded address for each ovitrap with Google maps. Paper towels were individually transported inside identified medium sized (17.7 × 18.8 cm) freezer Ziploc® bags (SC Johnson Co., San Mateo Otzacatipan, México) to the Coordinación Regional of Programa Nacional de Manejo Integrado de Vectores in Puntarenas, Costa Rica, where eggs were counted under a 10× dissecting microscope. The counting was carried out under a dissecting microscope to ensure that eggs of *Limatus* spp., if present, were separated from *Aedes* spp. eggs, given the striking morphological differences between these two mosquito genera and the potential colonization of ovitraps by *Limatus durhami* Theobald, a species present in the study area (Calderón-Arguedas et al., 2009), and considering that *Limatus* spp. also oviposit on surfaces above the water line (Santos Neto & Azevedo Marques, 1996; Ceretti-Júnior et al., 2014; Carvalho et al., 2017). Briefly, *Limatus* spp. eggs have a shape similar to a boomerang (or elongated rhombus), as opposed to the elongated elliptical shape of *Aedes* spp. eggs (Santos Neto & Azevedo Marques, 1996). Other species in the studied area (Vargas, 1961; Calderón-Arguedas et al., 2009) that could potentially colonize ovitraps included *Culex* spp. and *Uranotaenia* spp., both of which oviposit in rafts and whose eggs are projected into the water surface (Harbach & Knight, 1978; Day, 2016), and *Toxorhynchites* spp., which oviposit single eggs, that are significantly larger than eggs from other mosquito genera and that are also projected into the water surface (Chadee et al., 1987). For further mosquito identification quality control, each week up to 100 eggs from 3 randomly chosen traps were allowed to hatch to verify the identity of the species collected. Throughout the study, eggs from each ovitrap location were checked at least once; all samples collected belonging to *Ae. aegypti.* In addition, Puntarenas peninsula is an area without current or historical records for the presence of *Aedes albopictus* (Skuse) (Futami et al., 2015). During this study, we did not find *Limatus* spp. eggs while examining and counting sampled eggs under the dissecting microscope.

### 2.3 Arboviral disease data

Weekly counts for Zika, dengue and chikungunya cases from Puntarenas county, for all the weeks of 2017 and 2018, were obtained from the weekly epidemiological bulletins published by the epidemic surveillance division (Vigilancia de la Salud) from Costa Rica’s Ministry of Health which are publicly available at: https://www.ministeriodesalud.go.cr/index.php/vigilancia-de-la-salud/boletines/enfermedades-de-transmision-vectorial-2017. These case counts include all clinically diagnosed dengue cases, of which a variable proportion, ensuring all presumed transmission clusters are sampled, are confirmed by PCR and/or serological methods by the Costa Rican National Reference Laboratory for viral infections (González Elizondo, 2018). All Zika and chikungunya cases are confirmed by laboratory methods (PCR and/or serology), before being consolidated and reported by the epidemic surveillance division at Costa Rica’s Ministry of Health. Independently of the moment when the final diagnostic is performed, cases are recorded for the week individuals attend health facilities with symptoms of an arboviral infection.

### 2.4 Remotely sensed data (temperature and vegetation growth)

We downloaded daily images for land surface temperature and emissivity (LST&E) with a spatial resolution of 1 km (MOD1101, Version 6) based on Moderate Resolution Imaging Spectroradiometer (MODIS) images (Wan et al., 2015). The images were re-scaled by multiplying each pixel by a factor of 0.02, and the resulting temperatures were transformed from °K to °C by subtracting 273 (Wan et al., 2015). Downloaded MODIS images can be seen in the supplementary online Video S1. We generated Landsat 8 land surface temperature images with a spatial resolution of 30 m using a script for online use with Google Earth Engine (Ermida et al., 2020). Downloaded Landsat 8 images can be seen in the supplementary online Video S2 and were available every 8 days.

Supplementary videos related to this article can be found at https://doi.org/10.1016/j.crpvbd.2021.100014

The following are the supplementary data related to this article:

**Video S1.** MODIS images considered in the analysis. At the top of each image there is a code were “MOD11A1” indicates the NASA products employed, followed by “LST” (land surface temperature)m this is followed by “Day” to indicate data were daily and “1 km” indicating the 1 km spatial resolution of the data and finally the year, indicated by 4 digits, and the day of the year indicated by 3 digits. In all images defective pixels were removed, and the legend on the right side of each panel indicates the temperature in degrees Celsius. Video S1

**Video S2.** Landsat 8 images considered to build the land surface temperature time series. At the top of each image there is a code were LC08 indicates that images come the NASA-USGS Landsat 8 mission, followed by a 6 digits number that indicates the position of each of the images from which the Puntarenas peninsula area was clipped. The final 8 digits indicate the date when the image was taken. Specifically, the first 4 digits indicate the year (2017 or 2018), the next two digits the month, and the last two digits represent the day of the month. In all images defective pixels were removed, and the legend on the right side of each panel indicates the temperature in degrees Celsius. Each pixel has a resolution of 30 m. Video S2

The following is the supplementary data related to this article:

**Video S3.** Sentinel 2 images considered to build the vegetation index time series. In the RGB (red, green and blue) images, the first 8 digits indicate the date when the image was acquired by the satellite, the first 4 digits indicating the year (2017 or 2018), the next 2 digits indicate the month, and the last 2 digits the day of the month. The remaining 7 digits indicate the code for the image from where the Puntarenas Peninsula area was clipped. In all images defective pixels were removed. Each pixel has a resolution of 10 m.

We also downloaded images for the Normalized Difference Vegetation Index (NDVI) and Enhanced Vegetation Index (EVI), both of which are considered proxies for vegetation growth (Pettorelli et al., 2005) and have been extensively used to study the ecology of mosquitoes (Hurtado et al., 2018b; Chaves et al., 2019; Poh et al., 2019; Rigg et al., 2019; Nguyen et al., 2020; Chaves & Friberg, 2021) and other insect vectors (Kitron et al., 1996). The images for vegetation indices were downloaded for the multiple spatial scales encompassed by MODIS, Landsat 8 and Sentinel 2. The MODIS NDVI and EVI images were processed every 16 days, have a spatial resolution of 250 m (MOD13Q1, version 6 and MYD13Q1, version 6) and are collected by Terra and Aqua satellites (Didan, 2015a, b). These images were re-scaled by dividing the values of each pixel by 10,000, thus obtaining normalized values that range between −1 and 1 (Pettorelli et al., 2005). We used data from Terra (MOD13Q1) and Aqua (MYD13Q1) satellites, to increase the number of observations used to estimate environmental time series. This also allowed us to increase the frequency of observations to every 8 days provided the delay between the images from each satellite for the study site.

We also used information from the red (RED, band 4) and near infrared (NIR, band 5) Landsat 8 bands, with a resolution of 30 m, downloaded with the Landsat 8 land surface temperature to estimate NDVI using the following equation (Vermote et al., 2016):

(1) $$NDVI=\frac{\left(NIR-RED\right)}{\left(NIR+RED\right)}$$

Adding information from the Landsat 8 blue band (BLUE, band 2), we estimated EVI using the following equation (Vermote et al., 2016):

(2) $$EVI=2.5*\frac{\left(NIR-RED\right)}{\left(NIR+6*RED-7.5*BLUE+1\right)}$$

The resulting images were used to estimate Landsat 8-based NDVI and EVI. Equations (1), (2) were also used to estimate NDVI and EVI using top of the atmosphere Sentinel 2 images, which have a resolution of 10 m and where BLUE is band 2, RED is band 4 and NIR is band 8 (Drusch et al., 2012). Sentinel 2 images for the study period were processed using the Google Earth Engine to remove pixels covered by clouds or whose values exceeded the theoretical bounding values of −1 to 1 (ESA, 2015). Downloaded Sentinel 2 images can be seen in the supplementary online Video S3, and were available every 3.4 ± 2.0 days.

Supplementary videos related to this article can be found at https://doi.org/10.1016/j.crpvbd.2021.100014

The following is the supplementary data related to this article:

**Video S3** Sentinel 2 images considered to build the vegetation index time series. In the RGB (red, green and blue) images, the first 8 digits indicate the date when the image was acquired by the satellite, the first 4 digits indicating the year (2017 or 2018), the next 2 digits indicate the month, and the last 2 digits the day of the month. The remaining 7 digits indicate the code for the image from where the Puntarenas Peninsula area was clipped. In all images defective pixels were removed. Each pixel has a resolution of 10 m.

To generate MODIS-based temperature, NDVI and EVI time series, each image was cropped using a polygon from the joint area of the 5 districts conforming the urban area of Puntarenas (Fig. 1) using the package “*raster*” in R (Brunsdon & Comber, 2015) and mean values were estimated for the selected pixels containing information. We repeated this process for the Landsat 8-based temperature images, and Landsat 8- and Sentinel 2-based NDVI and EVI images, but cropping only the area corresponding to the Puntarenas peninsula (Fig. 1). The difference in the cropped areas was due to three main reasons: (i) over 90% of the arboviral cases occur in the Puntarenas city area of Puntarenas county, with over 80% of the cases linked with activities in the Puntarenas peninsula area (Vigilancia de la Salud, 2019) and such area is more easily covered by the lower resolution MODIS images; (ii) traps were deployed in the Puntarenas peninsula area, at distances where the spatial resolution of Landsat 8 and Sentinel 2 images is more likely to detect differences in the local environments were traps were located (which had a mean ± standard deviation (SD) of 495 ± 311 m, Supplementary Fig. S1); and (iii) having multi-scale spatial images allowed us to test the hypothesis that both local and regional environmental factors might be involved in arbovirus transmission and *Ae. aegypti* oviposition. In addition, for MODIS and Landsat 8 temperature images we estimated time series for the SD and kurtosis, respectively, in the Puntarenas city and peninsula area, given the possibility that arboviral cases and egg counts are sensitive to patterns of variability in the environment, as predicted by Schmalhausen’s law, the biological principle stating that organisms follow both the mean and higher order moments, i.e. SD and kurtosis, of environmental variation (Chaves, 2017a).

The resulting time series were then smoothed using the loess algorithm for local polynomial regression fitting (Cleveland & Devlin, 1988) by performing a regression of the estimated temperature, NDVI or EVI as a function of the day when the images were acquired by the satellites. For the loess, we employed a neighborhood size of 10% of the data and second-degree polynomials.

The resulting smoothed time series were then used to obtain weekly estimates of NDVI and EVI, extrapolating values to the Monday of each week in the case of NDVI and EVI from MODIS, Landsat 8 and Sentinel 2 images, and also for land surface temperatures, including mean, SD and kurtosis from Landsat 8 images. However, for MODIS-based temperatures, we estimated weekly average, SD and kurtosis from Tuesday to Monday, using the daily temperature time series.

All MODIS and Landsat 8 images were courtesy of the NASA Land Processes Distributed Active Archive Center (LP DAAC) and the United States Geological Survey (USGS)/Earth Resources Observation and Science (EROS) Center (Sioux Falls, South Dakota). Copernicus Sentinel 2 data were processed, and provided free of charge, by the European Space Agency (ESA). All MODIS images were downloaded from the LP DAAC server (NASA, 2018) using the package *MODIStsp* in R (Busetto & Ranghetti, 2016). Landsat 8 and Sentinel 2 images were retrieved from the Google Earth Engine Dataset Catalog (Google, 2020).

### 2.5 Statistical analysis

Our statistical analysis had four main components.

#### 2.5.1 Comparison of land surface temperature (LST) time series estimates with MODIS and Landsat 8 images

We examined LST time series estimated with MODIS (Supplementary Fig. S2A) and Landsat 8 (Supplementary Fig. S2B) using the Pearson’s correlation coefficient (Zar, 1998). We also compared MODIS and Landsat 8 data examining their coefficient of variation (CV). The CV, formally defined as the ratio between the SD and the mean of a variable (Stearns, 1981), was estimated for each observation in the MODIS (Supplementary Fig. S2C) and Landsat 8 (Supplementary Fig. S2D) time series. Then we assessed any potential bias in the estimation of time series observations related to the number of available pixels by performing a linear regression of the LST CV as a function of the number of pixels used to estimate LST. We used MODIS images for the Puntarenas city area (Supplementary Fig. S2E) and Landsat 8 images (Supplementary Fig. S2F) for the Puntarenas peninsula area when estimating the regressions.

#### 2.5.2 Time series modeling

We studied the association between *Ae. aegypti-*borne arboviruses (Zika, chikungunya and dengue) and *Ae. aegypti* egg counts and remotely sensed environmental variables using the general framework of seasonal autoregressive (SAR) models (Shumway & Stoffer, 2011). SAR models have the general form:

(3) $$X_{t}=\mu +\sum_{j=1}^{lmax}\varphi_{j}\left(X_{t-j}-\mu \right)+\sum_{s>lmax}^{smax}\varphi_{s}\left(X_{t-s}-\mu \right)-\sum_{j=1}^{lmax}\sum_{s>lmax}^{smax}\varphi_{j}\varphi_{s}\left(X_{t-j-s}-\mu \right)+\sum_{i=1}^{k}\beta_{i}Cov{\left(i\right)}_{t-m}+\varepsilon_{t}$$

where $X_{t}$ is the focal variable, i.e., arboviral cases or mean number of eggs in this study, observed at time *t*, $X_{t}\;$is a function of itself at time *t-j*, and at time *t-s*, where *j* and *s* are the time lags for the “autoregressive” and “seasonal” parameters ($\varphi_{j\;}\text{ ​and ​}\varphi_{s},$ respectively) that account for autoregressive and cyclical patterns in the time series of the focal variable studied, and *Cov(i)* indicates covariates, which are related to the focal variable under study according to parameters $\beta_{i}$. The SAR models can have any *k* number of covariates, as long as this number is shorter than the time series length, minus the number of seasonal and autoregressive parameters plus one, μ is the mean of the time series, and $\varepsilon_{t}\;$is the error, which is assumed to be normal, independent and identically distributed (Shumway & Stoffer, 2011). To develop SAR models, we started by the identification of the maximum time lags for the autoregressive and seasonal components of the model. For lag identification we employed autocorrelation (ACF) and partial autocorrelation (PACF) functions which show the correlation of time series observations at different time lags considering, respectively, the full time series or only consecutive lags (Hoshi et al., 2014). Based on information from these functions, a null SAR model was fitted and used to pre-whiten time series of covariates whose association is then evaluated by examining cross-correlation functions (CCFs) between the covariate time series and the focal time series. This analysis allows the identification of lags at which covariates are correlated with the studied variables. Pre-whitening was used to avoid the spurious identification of significant lags of association that emerge from time series having similar autocorrelation structures (Hoshi et al., 2017).

Models for the weekly combined case count of dengue, chikungunya and Zika considered as potential covariates the median, the SD and kurtosis of weekly collected eggs across all ovitraps, the mean of remotely sensed temperature, NDVI and EVI at the different spatial resolutions considered in this study. We also studied the median number of weekly collected eggs as a function of the mean of remotely sensed temperature, NDVI and EVI at the different spatial resolutions considered in this study. In both cases we considered the MODIS-based time series estimated for the whole Puntarenas city and the Landsat 8- and Sentinel 2-based time series estimated for the Puntarenas peninsula. This was done to test if environmental conditions specific to the area where most transmission is assumed to occur, i.e., the Puntarenas peninsula, were associated with transmission recorded for the larger Puntarenas city area in models for arboviral cases. Similarly, in models for *Ae. aegypti* egg counts we considered the MODIS-based environmental time series for the Puntarenas city area to test if environmental phenomena occurring at larger spatial scales could influence *Ae. aegypti* oviposition dynamics recorded in the Puntarenas peninsula.

When fitting the models, all selected covariates were demeaned to ease their interpretation in terms of changes above or below their means (Chaves et al., 2018). We selected the best models through the minimization of the Akaike Information Criterion (AIC), a metric that trades-off model goodness-of-fit and parameter number, employing a strategy of backward elimination, where models are simplified by comparing “nested” models with the same number of parameters that are not significantly different from the “parent” model that considered all variables that are left out in each of the simplified “nested” models (Chaves, 2016; Chaves & Moji, 2018), or until there were no significant differences through a Chi-square likelihood ratio test between the model minimizing AIC and simplified versions of such model (Chaves et al., 2019).

#### 2.5.3 Synchrony analysis

We performed a synchrony analysis for *Ae. aegypti* oviposition and environmental variables estimated with Landsat 8 and Sentinel 2 images. This analysis was carried out to understand if concerted fluctuations in eggs/ovitrap during the study period followed similar changes in temperature and the vegetation indices that we studied. For the analysis, we employed a Mantel correlogram with inference based on a Monte Carlo test, whose null hypothesis is that synchrony across the range of distances separating sampling locations is equal to a global average, unless more extreme (Chaves, 2017b; Chaves et al., 2020b). For the analysis, we only considered up to 50% of the spatial extent of the data to avoid spurious results at long distances due to small sample size (Gouhier & Guichard, 2014), but estimated regional synchrony considering samples from all the georeferenced ovitraps that had, at most, one missing observation. To estimate the synchrony, we used the time series from the 44 ovitrap locations that did not have (*n* = 27) or had at most one missing observation (*n* = 17), which were inputted by taking the average of the two immediate observations, i.e. one time step before and after the missing observation, in order to increase the power for statistical inference (Gouhier & Guichard, 2014). We also estimated synchrony for environmental variables at the pixels that contained the ovitraps considered in the oviposition synchrony analysis. For this we extracted Land Surface Temperature (LST), NDVI and EVI data from Landsat 8 images, and NDVI and EVI data from Sentinel 2 images, at the pixels containing the 44 ovitraps used for the *Ae. aegypti* oviposition synchrony analysis. We inputted missing values for the resulting environmental time series following the procedure described in the time series modeling section. The 44 ovitraps used for this analysis did not include any of the two ovitraps placed outside the houses.

#### 2.5.4 Spatial analysis

We tested whether mean number of *Ae. aegypti* egg counts at georeferenced ovitraps (*n* = 81, after excluding two locations whose remotely sense data was defective and nine that were impossible to be georeferenced, among which were the two placed outside the housing premises) were correlated with Landsat 8 (LST, NDVI, EVI) and Sentinel 2 (NDVI and EVI) remotely sensed data. We employed a linear regression described by the following equation:

(4) $$\;Y_{i}=\mu +\sum_{j=1}^{k}\beta_{j}Cov{\left(j\right)}_{i}+\varepsilon_{i}$$

where $Y_{i}$ is the mean number of *Ae. aegypti* eggs, through the study period, at ovitrap location *i*, *Cov(j)* indicates the mean of remotely sensed environmental covariates, which are related to the mean number of *Ae. aegypti* eggs according to parameters $\beta_{j}$, μ is the intercept, and $\varepsilon_{i}\;$is the error, which is assumed to be normal, independent and identically distributed (Faraway, 2004). Given that observations used to estimate the mean number of *Ae. aegypti* eggs at the ovitrap locations were not homogeneous during the study period, as a few traps were vandalized or contents accidently removed by household residents, we used error weights proportional to the number of trap-weeks used to estimate the means to accurately represent sampling effort during parameter estimation (Faraway, 2004). We then proceeded with model selection based on the minimization of the AIC, following the same steps already described for the time series models. We tested the best model for nonlinearities in the association with the selected covariates, and if any of the associations was non-linear, we proceeded with fitting a generalized additive model (Venables & Ripley, 2002) that used a smoothed function for the covariates whose association with the mean number of *Ae. aegypti* eggs was nonlinear. For the generalized additive model, we also used error weights proportional to the sampling effort at each ovitrap location.

Finally, we performed a Moran’s I test on the best model residuals, a test with a null hypothesis that a variable is spatially independent (Brunsdon & Comber, 2015). For the Moran’s I test we generated a spatial weights matrix linking ovitrap sampling locations within a distance of 145 m, the largest minimum distance between any two ovitrap sampling locations. Once neighbors were identified, weights were made proportional to the number of neighbors for each sampling location (Brunsdon & Comber, 2015).

## 3 Results

### 3.1 Data summary statistics

**Fig. 4:**
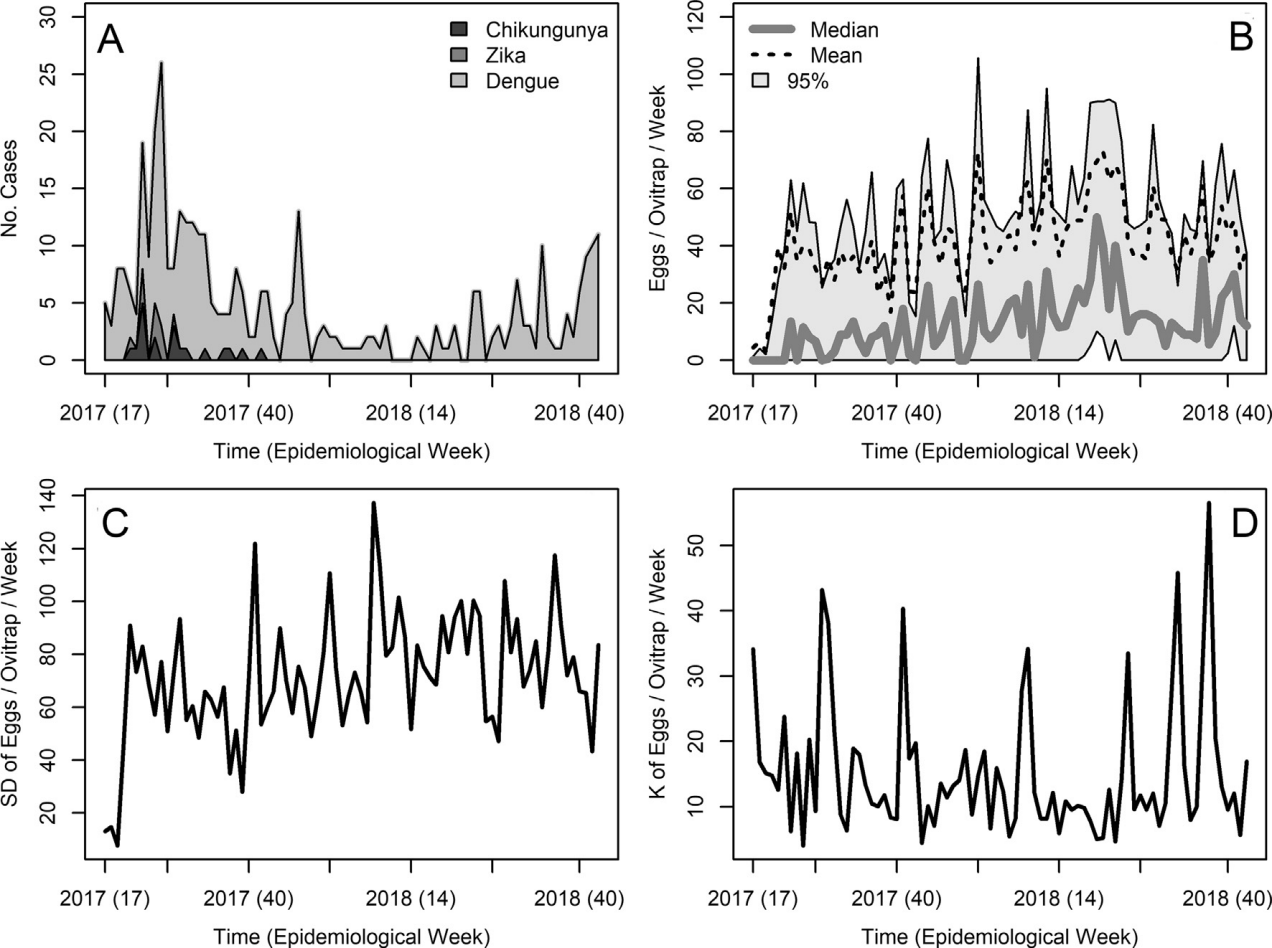
Arboviral cases and mosquito time series. **A** Weekly counts of *Aedes aegypti*-borne arboviral cases in Puntarenas County. **B** Weekly distribution of *Ae. aegypti* egg counts across the ovitrap sampling locations. **C** Weekly standard deviation (SD) of *Ae. aegypti* egg counts across the ovitrap sampling locations. **D** Weekly kurtosis (K) of *Ae. aegypti* egg counts across the ovitrap sampling locations. Raw data including individual time series for each ovitrap sampling location are presented in Supplementary Fig. S3 at the journal website. See the inset legends for further details

A total of 381 cases of *Ae. aegypti*-borne arbovirus cases were recorded in Puntarenas county during the ovitrap monitoring period, i.e. from epidemiological week 17 of 2017 to epidemiological week 44 of 2018 (Fig. 4A). Of these, 11 were Zika infections, 19 were chikungunya infections and the remaining 351 cases were due to dengue infections (Fig. 4A). During the study period we counted a total of 291,369 *Ae. aegypti* eggs, over a total sampling effort of 7,360 ovitrap weeks. A total of 278 times (3.78% of the total sampling effort), we were unable to collect the paper towels with eggs from ovitraps due to diverse reasons, mainly accidental disturbances from residents, and very few instances of vandalism (only 5 ovitraps needed to be replaced during the study duration). Ovitraps had no eggs 39.56% of the times they were sampled, i.e. 2,912 times, with a weekly average (±SD) of ovitraps without eggs of 36.40 ± 10.91, weekly ranging from 14 to 62 ovitraps without eggs across the studied period. The average number of eggs (±SD) per ovitrap/week was 41.14 ± 75.94 with weekly trap counts ranging from 0 to 1,000 eggs (Fig. 4B). Raw data for individual ovitraps are presented in Supplementary Fig. S3. In general, the mean number of eggs for ovitraps was larger than the median, indicating oviposition was clustered at few ovitrap locations during the study period (Fig. 4B). The SD (Fig. 4C) and kurtosis (Fig. 4D) of eggs/ovitrap/week had regular peaks during the study period, which did not seem to coincide with peaks in arbovirus transmission (Fig. 4A) or egg counts (Fig. 4B).

### 3.2 Comparison of land surface temperature (LST) time series estimates with MODIS and Landsat 8 images and remotely sensed data seasonality

**Fig. 5:**
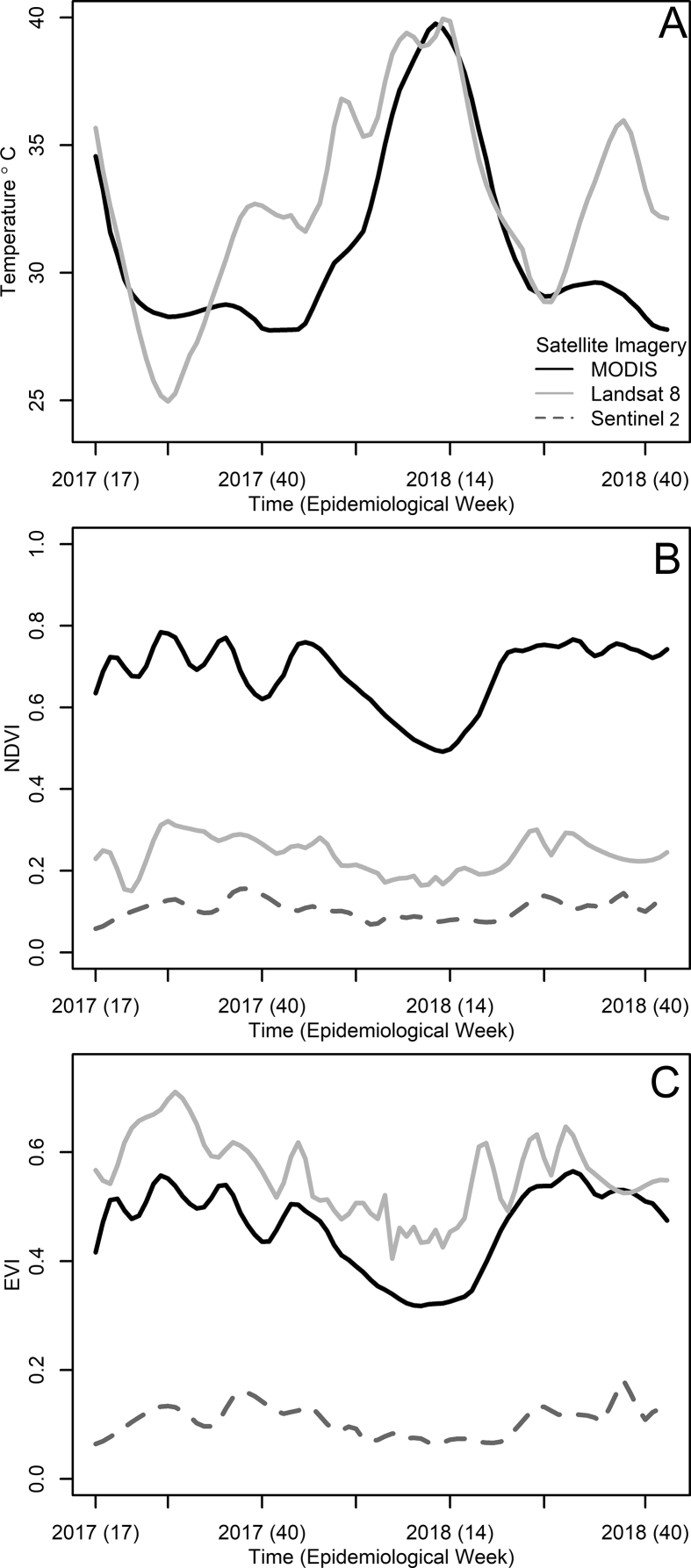
Remotely sensed environmental time series. **A** Weekly mean land surface temperature derived from satellite images. **B** Weekly NDVI derived from satellite images. **C** Weekly EVI derived from satellite images. Temperature data originate from daily MODIS images, and Landsat 8 images collected every 8 days. NDVI and EVI time series also included Sentinel 2 data, which were collected every 3.4 ± 2.0 days (mean ± SD). For guidance, please refer to the inset legend of panel **A**. Raw and weekly smoothed data used to estimate these time series are presented in Supplementary Fig. S2 for MODIS and Landsat 8 land surface temperature, and raw data for the NDVI and EVI, derived from MODIS, Landsat 8 and Sentinel 2 are provided in Supplementary Fig. S5

MODIS and Landsat 8-based Land Surface Temperature showed a clear seasonal pattern where temperature peaks were observed during the dry season of Puntarenas, weeks 1 to 16 of 2018 (Fig. 5A, raw MODIS and Landsat 8 data presented, respectively, in Supplementary Figs. S2A and S2B). These time series had a low CV (around 7%) for both MODIS (Supplementary Fig. S2C) and Landsat 8 (Supplementary Fig. S2D), meaning that pixel had a variability of up to 0.7 °C for each 10 °C of mean temperature in the areas over which the MODIS (Puntarenas city) and Landsat 8 (Puntarenas peninsula) time series were estimated. The estimates in the time series also had a very low sensitivity to the number of pixels considered for area estimations. Estimates, on average, only decreased on the order of 10^−5^ for MODIS (Supplementary Fig. S2E) and 10^−6^ for Landsat 8 (Supplementary Fig. S2F) for each pixel not considered on the area estimations. Interestingly, both time series, land surface temperature from MODIS and Landsat 8, were highly correlated (Pearson’s $\hat{r}$ = 0.734) despite the difference in resolution and areas over where these temperature time series were estimated. The MODIS-based temperature SD time series (Supplementary Fig. S4A) had a seasonal pattern different to its mean value, as the SD peaked during the dry season of Puntarenas. However, no clear patterns were observed for the MODIS-based temperature kurtosis time series (Supplementary Fig. S4B) which seemed to increase randomly, becoming more leptokurtic, i.e. with high kurtosis meaning temperature was more variable towards the extremes than around the mean of the distribution presented in Fig. 5A. The Landsat 8-based temperature SD (Supplementary Fig. S4C) and kurtosis (Supplementary Fig. S4D) time series had patterns similar to the ones observed for the MODIS-based time series.

Seasonality in NDVI (Fig. 5B) and EVI (Fig. 5C) were opposite to the seasonality observed in temperature, as both vegetation indices decreased when temperature reached peak values. For the study period, MODIS-based NDVI values were higher than those observed for Landsat 8 and Sentinel 2 (Fig. 5B). Meanwhile, MODIS- and Landsat 8-based EVI values were very similar, and higher than values generated with Sentinel 2 images (Fig. 5C). Raw data for MODIS NDVI (Supplementary Fig. S5A) and EVI (Supplementary Fig. S5B), Landsat 8 NDVI (Supplementary Fig. S5C) and EVI (Supplementary Fig. S5D), Sentinel 2 NDVI (Supplementary Fig. S5E) and EVI (Supplementary Fig. S5F) also showed a marked seasonal pattern, where both indices decreased during weeks 1 to 16 of 2018, suggesting the seasonality was not an artifact of smoothing the time series.

### 3.3 Time series analysis

**Fig. 6:**
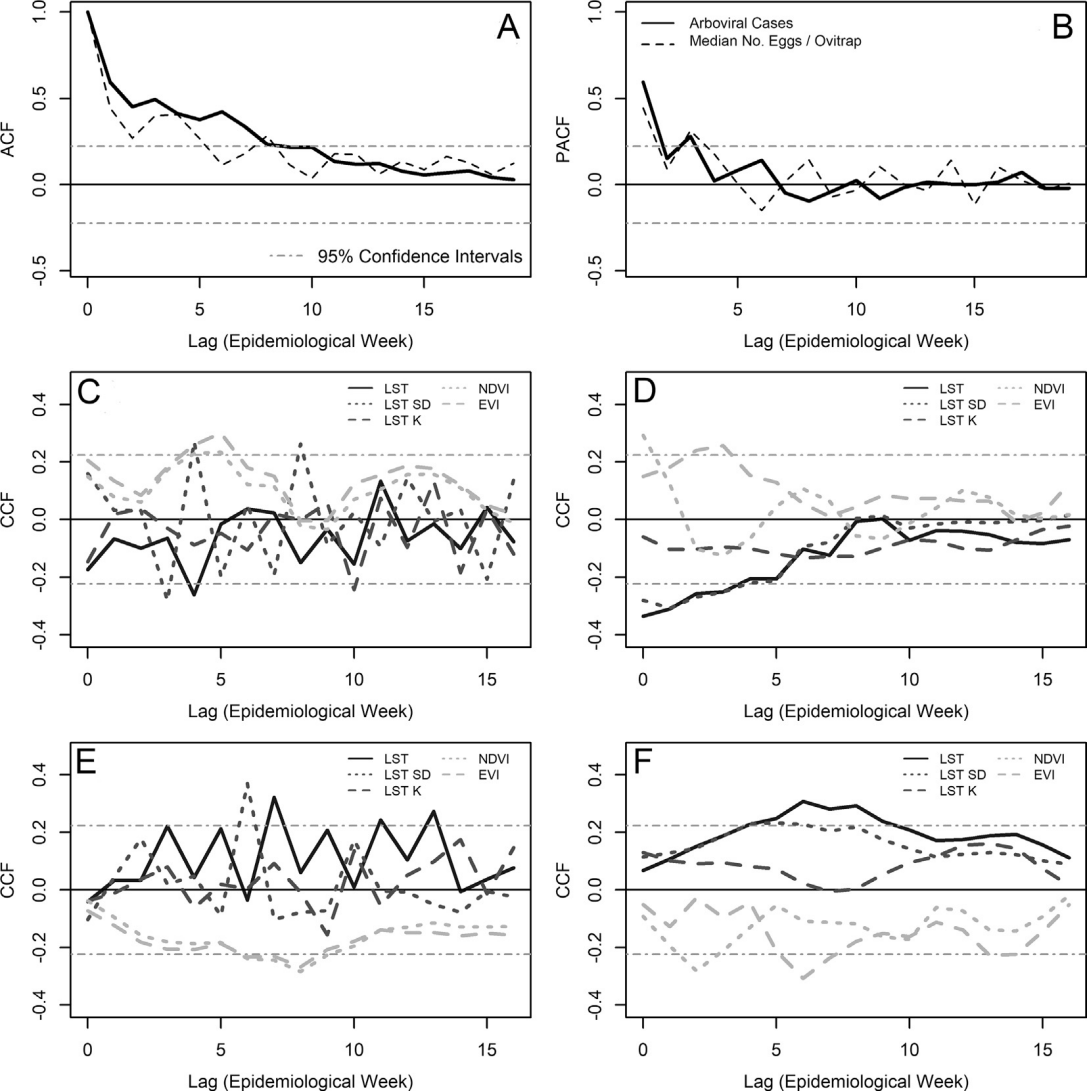
Correlation functions. **A** Autocorrelation (ACF) and **B** partial autocorrelation function (PACF) for arboviral cases and median number, per ovitrap, of *Aedes aegypti* egg counts weekly time series. Lines are identified in the inset legend of panel **B. C** Cross-correlation function (CCF) between weekly arboviral cases and MODIS-based environmental variables, including land surface temperature, NDVI and EVI mean values, and also the standard deviation (SD) and kurtosis (K) of land surface temperature. **D** CCF between weekly arboviral cases and Landsat 8-based environmental variables, including land surface temperature, NDVI and EVI mean values, and also the SD and kurtosis of land surface temperature. **E** CCF between median *Ae. aegypti* egg counts and MODIS-based environmental variables, including land surface temperature, NDVI and EVI mean values, and also the SD and kurtosis (K) of land surface temperature. **F** CCF between median *Ae. aegypti* egg counts and Landsat 8-based environmental variables, including land surface temperature, NDVI and EVI mean values, and also the SD and kurtosis (K) of land surface temperature. In all panels the dot-dashed 95% confidence interval lines (see panel **A** for reference) indicate that correlations within the area are expected by chance. This means that peaks outside the 95% confidence interval lines are the time lags at which the number of arboviral cases (or median egg counts) was associated with itself in the ACF and PACF plots, or with the covariates in the CCF plots

The inspection of the ACFs (Fig. 6A) and PACFs (Fig. 6B) suggested that *Ae aegypti*-borne arboviral cases and *Ae. aegypti* median egg counts followed first order seasonal autoregressive processes with a 3-week period. This means that observations, for both cases and egg counts, at any given time were associated with observations with a lag of one and three weeks. Using this information for these two variables, a null model with the following general form was fitted:

(5) $$X_{t}=\mu +\varphi_{1}\left(X_{t-1}-\mu \right)+\varphi_{3}\left(X_{t-3}-\mu \right)-\varphi_{1}\varphi_{3}\left(X_{t-4}-\mu \right)+\varepsilon_{t}$$

This was then used to pre-whiten the covariate time series in order to select lags at which MODIS-based (Fig. 6C) and Landsat 8-based (Fig. 6D) variables were associated with arbovirus cases according to their CCFs. We used this information to build a full time series model for arboviral cases, which after a process of model selection by backward elimination (Table 1) resulted in a first-order seasonal autoregressive model. The selected model showed that number of cases had a nonlinear response to changes in MODIS-based temperature SD increasing 1 case for each 2 units of SD increase with a 4-week lag, while decreasing almost 1 case for each 2 units of MODIS-based SD increase with a 3-and 4-week lag and decreasing up to five cases for each Landsat 8 temperature SD unit increase with a 1-week lag. Arboviral cases increased around 4 cases for each 0.1 units of Landsat 8-based NDVI increase during the same week cases were recorded (Table 2). Arboviral cases were not significantly associated with the median, SD or kurtosis of *Ae. aegypti* egg counts (Supplementary Fig. S6A) nor with Sentinel 2-based NDVI and EVI (Supplementary Fig. S6B).

**Table 1:** Model selection for the best time series model explaining counts of arbovirus cases at Puntarenas City, Costa Rica

Variables (Lag)	AIC
Null model: Autoregressive (1), Seasonal Autoregressive (3)	447.94
Full model: Autoregressive (1), Seasonal Autoregressive (3), MODIS-Temperature (4), MODIS-SD Temperature (3), MODIS-SD Temperature (4), MODIS-EVI (5), MODIS-NDVI (5), Landsat 8-Temperature (0), Landsat 8-SD temperature (1), Landsat 8-NDVI (0), Landsat 8-EVI (4)	436.08
Autoregressive (1), Seasonal Autoregressive (3), MODIS-SD Temperature (3), MODIS-SD Temperature (4), MODIS-EVI (5), MODIS-NDVI (5), Landsat 8-Temperature (0), Landsat 8-SD temperature (1), Landsat 8-NDVI (0), Landsat 8-EVI (4)	434.27
Autoregressive (1), Seasonal Autoregressive (3), MODIS-SD Temperature (3), MODIS-SD Temperature (4), MODIS-EVI (5), MODIS-NDVI (5), Landsat 8-Temperature (0), Landsat 8-SD temperature (1), Landsat 8-NDVI (0), Landsat 8-EVI (4)	432.52
Autoregressive (1), Seasonal Autoregressive (3), MODIS-SD Temperature (3), MODIS-SD Temperature (4), MODIS-EVI (5), Landsat 8-Temperature (0), Landsat 8-SD temperature (1), Landsat 8-NDVI (0), Landsat 8-EVI (4)	432.07
Autoregressive (1), Seasonal Autoregressive (3), MODIS-SD Temperature (3), MODIS-SD Temperature (4), MODIS-EVI (5), Landsat 8-Temperature (0), Landsat 8-SD temperature (1), Landsat 8-NDVI (0)	431.56
Autoregressive (1), Seasonal Autoregressive (3), MODIS-SD Temperature (3), MODIS-SD Temperature (4), Landsat 8-Temperature (0), Landsat 8-SD temperature (1), Landsat 8-NDVI (0)	**431.29**

*Notes*: AIC indicates the Akaike information criterion for each model. AIC is minimized by the best model, which is presented in bold. Lag indicates the time lag (in epidemiological weeks) for the correlation between arbovirus case number and the environmental variables considered.

**Table 2:** Parameter estimates for the best time series model explaining counts of arbovirus cases at Puntarenas City, Costa Rica

Parameter	Estimate ± SE
Mean	6.676 ± 6.166^a^
Autoregressive (1-week lag)	0.416 ± 0.107^a^
Seasonal autoregressive (3-weeks lag)	0.285 ± 0.114^a^
MODIS-based SD of temperature with 3-weeks lag	−0.468 ± 0.226^a^
MODIS-based SD of temperature with 4-weeks lag	0.488 ± 0.230^a^
Landsat 8-based SD of temperature with 1-week lag	−4.869 ± 1.766^a^
Landsat 8-based NDVI without a time lag	38.807 ± 15.426^a^
Variance error	10.46

*Abbreviation*: SE, standard error.

a Statistically significant (*P* <0.05).

The CCFs of median number of *Ae. aegypti* eggs per ovitrap were not significantly associated with Sentinel 2-based NDVI and EVI (Supplementary Fig. S6C). In contrast, the median number of *Ae. aegypti* eggs per ovitrap was significantly associated with several MODIS-based (Fig. 6E) and Landsat 8-based (Fig. 6F) environmental variables presented in the full model in Table 3. After the process of model selection, the seasonal component was not significant (Table 3) and the median of eggs increased by approximately three units for each unit of MODIS-based temperature SD, while decreasing approximately 7 units for each 0.1 increase of Landsat 8-based NDVI, both variables having their impact on median egg number with a lag of 6 weeks (Table 4). The ACF (Supplementary Fig. S7A), PACF (Supplementary Fig. S7B) and CCFs with MODIS (Supplementary Fig. S7C) and Landsat 8 (Supplementary Fig. S7D) of the mean number of *Ae. aegypti* per ovitrap were similar to the ones observed for the median number of *Ae. aegypti* egg counts per ovitrap.

**Table 3:** Model selection for the best time series model explaining the median number of *Aedes aegypti* eggs per ovitrap at Puntarenas City, Costa Rica

Variables (Lag)	AIC
Null model: Autoregressive (1), Seasonal Autoregressive (3)	588.92
Full model: Autoregressive (1), Seasonal Autoregressive (3), MODIS-Temperature (7), MODIS-SD Temperature (6), MODIS-NDVI (8), MODIS-EVI (8), Landsat 8-Temperature (6), Landsat 8-SD Temperature (5), Landsat 8-NDVI (2), Landsat 8-EVI (6)	574.96
Autoregressive (1), Seasonal Autoregressive (3), MODIS-Temperature (7), MODIS-SD Temperature (6), MODIS-NDVI (8), MODIS-EVI (8), Landsat 8-Temperature (6), Landsat 8-NDVI (2), Landsat 8-EVI (6)	573.25
Autoregressive (1), Seasonal Autoregressive (3), MODIS-SD Temperature (6), MODIS-NDVI (8), MODIS-EVI (8), Landsat 8-Temperature (6), Landsat 8-NDVI (2), Landsat 8-EVI (6)	571.62
Autoregressive (1), Seasonal Autoregressive (3), MODIS-SD Temperature (6), MODIS-EVI (8), Landsat 8-Temperature (6), Landsat 8-NDVI (2), Landsat 8-EVI (6)	570.99
Autoregressive (1), Seasonal Autoregressive (3), MODIS-SD Temperature (6), Landsat 8-Temperature (6), Landsat 8-NDVI (2), Landsat 8-EVI (6)	570.03
Autoregressive (1), Seasonal Autoregressive (3), MODIS-SD Temperature (6), Landsat 8-Temperature (6), Landsat 8-EVI (6)	569.90
Autoregressive (1), Seasonal Autoregressive (3), MODIS-SD Temperature (6), Landsat 8-EVI (6)	**569.90**^a^
Autoregressive (1), MODIS-SD Temperature (6), Landsat 8-EVI (6)	571.18^a^

*Notes*: AIC indicates the Akaike information criterion for each model. AIC is minimized by the best model, which is presented in bold. Lag indicates the time lag (in epidemiological weeks) for the correlation between arbovirus case number and the environmental variables considered.

a Selected as best model due to the lack of a significant difference with the model minimizing AIC, through a Chi-square likelihood ratio test (χ^2^ = 1.641, *df*  = 1, *P*  >  0.200).

**Table 4:** Parameter estimates for the best time series model explaining the median number of *Aedes aegypti* eggs per ovitrap at Puntarenas City, Costa Rica

Parameter	Estimate ± SE
Mean	51.074 ± 10.775^a^
Autoregressive (1-week lag)	0.400 ± 0.107^a^
MODIS-based SD of temperature with 6-weeks lag	2.820 ± 0.609^a^
Landsat 8-based EVI with 6-weeks lag	−69.462 ± 19.297^a^
Variance error	65.02

*Abbreviation*: SE, standard error.

a Statistically significant (*P*  <  0.05).

### 3.4 Synchrony

**Fig. 7:**
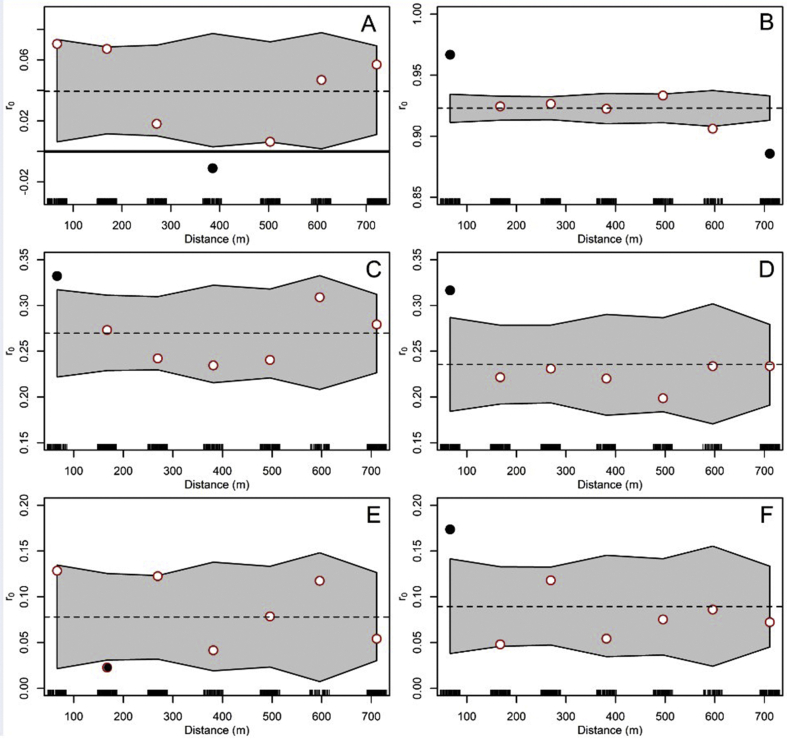
Synchrony. **A** Weekly *Aedes aegypti* egg counts. **B** Landsat 8-based land surface temperature. **C** Landsat 8-based NDVI. **D** Landsat 8-based EVI. **E** Sentinel 2-based NDVI. **F** Sentinel 2-based EVI. Dots are average synchrony estimates for a given distance and the gray area indicates the 95% confidence intervals of the synchrony. For reference, the global (or average) synchrony (*r*_*0*_) is presented as a black dashed line, and the black solid line indicates when synchrony is equal to zero. Filled circles represent synchrony estimates that are significantly different from the average synchrony (*P*  <0.05), while empty circles are not statistically different from the average synchrony estimate (*P*  > 0 .05)

*Aedes aegypti* oviposition synchrony patterns are presented in Fig. 7A. Overall, oviposition synchrony was very low, with a regional synchrony of 0.04, which although statistically significant was very close to 0, i.e. a total lack of synchrony. The observed synchrony pattern is also suggestive of a travelling wave, as suggested by the 7 point estimates shown in Fig. 7A, where synchrony changes from positive to negative, around 400 m (where synchrony is significantly different from the regional estimate, as indicated by the black dot in Fig. 7A), suggesting patterns observed in Fig. 4B reflect a heterogeneous, or with dynamic spatiotemporal clusters, oviposition through time and space, also suggesting dynamic changes in the entomological exposure to *Ae. aegypti* in Puntarenas city. Landsat 8-based temperature (Fig. 7B) NDVI (Fig. 7C) and EVI (Fig. 7D) were significantly more synchronous than *Ae. aegypti* oviposition, with synchrony below 100 m being significantly higher than the regional values, suggesting these variables were not associated with *Ae. aegypti* oviposition in Puntarenas peninsula. Sentinel 2-based NDVI (Fig. 7E) synchrony significantly decreased at distances around 170 m when compared with the regional synchrony, while Sentinel 2-based EVI (Fig. 7F) had a pattern similar to the one observed for Landsat 8-based environmental variables with synchrony below 100 m being significantly higher than the regional values. Sentinel 2-based vegetation indices had low regional synchrony values similar to the ones observed for oviposition, suggesting oviposition synchrony might be driven by finely-grained environmental phenomena. For example, NDVI (Fig. 7E) decreased below the regional synchrony at distances of 170 m, the pattern most similar to what was observed for oviposition synchrony (Fig. 7A).

### 3.5 Spatial models

**Fig. 8:**
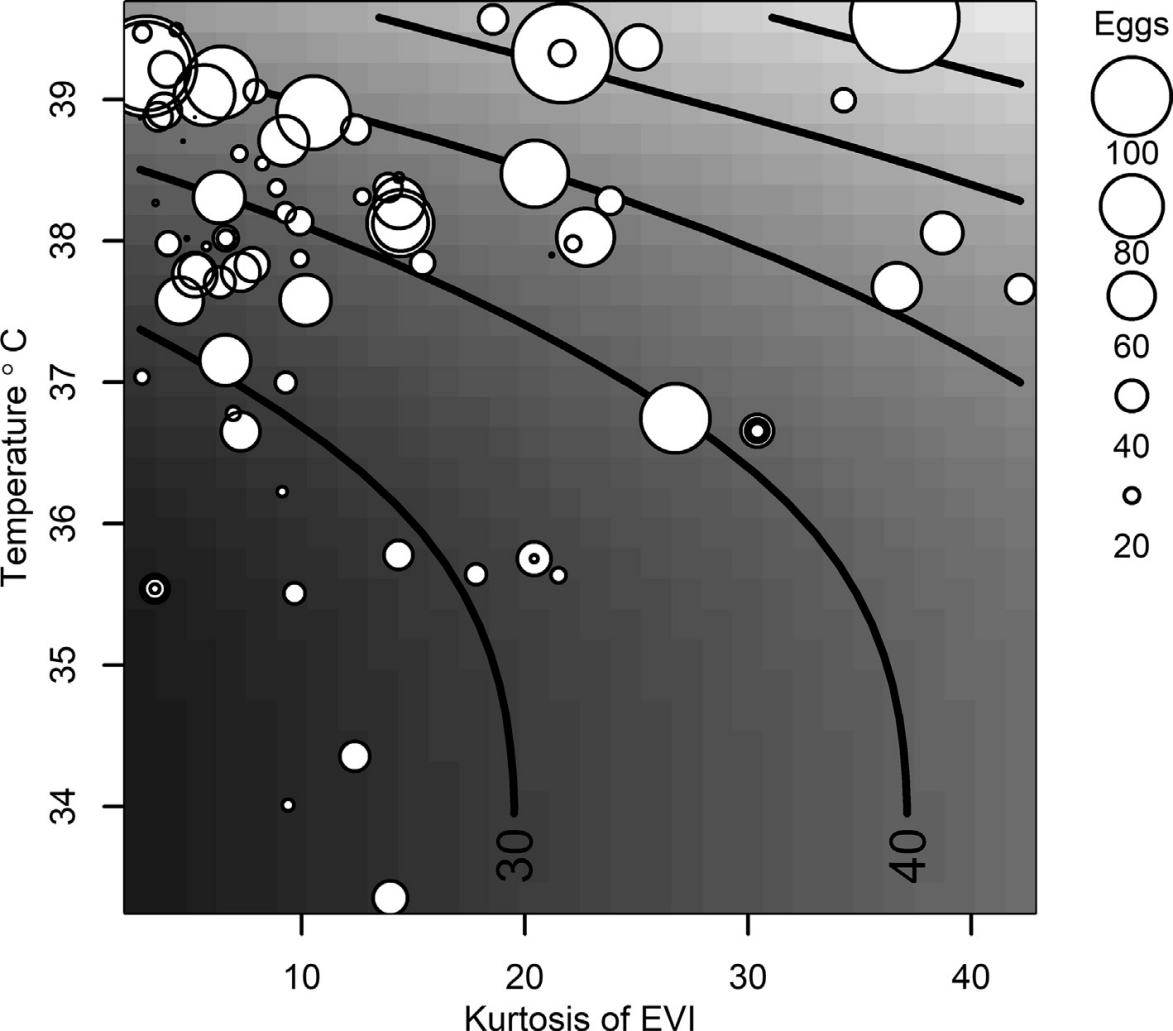
Fitted surface from the best Gaussian generalized additive model explaining the mean number of *Aedes aegypti* egg counts per ovitrap as a function of Landsat 8-based EVI kurtosis and land surface temperature. Contour lines indicate the expected mean number of *Ae. aegypti* eggs per ovitrap. Contour lines were drawn in increasing values of 10 and observed values are represented by circles proportional to the mean number of eggs per ovitrap

Selection for the best spatial model (Table 5) allowed us to identify Landsat 8-based EVI kurtosis and temperature as variables significantly associated with the mean number of *Ae. aegypti* eggs per ovitrap. The relationship with Landsat 8-based EVI kurtosis (*LEVIK*) was linear, with the number increasing by 3 eggs for each unit of kurtosis increase (Table 6), suggesting that leptokurtic vegetation changes were associated with the oviposition of more eggs. However, the association with temperature (*T*) was nonlinear and we fitted a Gaussian generalized additive model described by the following equation:

(6) $$Y_{i}=\mu +\beta LEVIK+s\left(T\right)+s\left(T,LEVIK\right)+\varepsilon_{i}$$

where *s*( ) denotes a smoothed function and parameters and assumptions were presented when defining Equation (4). The fitted surface is shown in Fig. 8, where it can be observed that the mean number of eggs increases with both *T* and *LEVIK*. The model surface shows that number of eggs increase at a rate of approximately 10 additional eggs for each °C above 36 °C, with temperature having a minimal impact on the mean number of *Ae. aegypti* eggs when below 36 °C. Finally, lack of significance for the Moran’s I statistic (Table 6) supports that assumptions of spatial independence for the error were met, and therefore inferences for the model presented in Table 6 are valid.

**Table 5:** Model selection for the best spatial Gaussian generalized additive model explaining the median number of *Aedes aegypti* eggs per ovitrap at Puntarenas City, Costa Rica

Variables	AIC
Full model: LST, LNDVI, LEVI, SNDVI, SEVI, LSTSD, LNDVISD, LEVISD, SNDVISD, SEVISD, LSTK, LNDVIK, LEVIK, SNDVIK, SEVIK	566.91
LST, LNDVI, LEVI, SNDVI, SEVI, LSTSD, LNDVISD, LEVISD, SNDVISD, SEVISD, LSTK, LNDVIK, LEVIK, SEVIK	564.91
LST, LEVI, SNDVI, SEVI, LSTSD, LNDVISD, LEVISD, SNDVISD, SEVISD, LSTK, LNDVIK, LEVIK, SEVIK	562.91
LST, LEVI, SNDVI, SEVI, LSTSD, LNDVISD, LEVISD, SNDVISD, SEVISD, LSTK, LNDVIK, LEVIK	560.92
LST, SNDVI, SEVI, LSTSD, LNDVISD, LEVISD, SNDVISD, SEVISD, LSTK, LNDVIK, LEVIK	558.95
LST, SNDVI, SEVI, LSTSD, LNDVISD, SNDVISD, SEVISD, LSTK, LNDVIK, LEVIK	557.00
LST, SNDVI, SEVI, LSTSD, LNDVISD, SEVISD, LSTK, LNDVIK, LEVIK	555.10
LST, SEVI, LSTSD, LNDVISD, SEVISD, LSTK, LNDVIK, LEVIK	553.29
LST, SEVI, LSTSD, SEVISD, LSTK, LNDVIK, LEVIK	551.47
LST, SEVI, LSTSD, LSTK, LNDVIK, LEVIK	549.75
LST, LSTSD, LSTK, LNDVIK, LEVIK	547.93
LST, LSTSD, LNDVIK, LEVIK	546.31
LST, LSTSD, LEVIK	545.22
**LST, LEVIK**	**544.25**

*Notes*: AIC indicates the Akaike information criterion for each model. AIC is minimized by the best model, which is presented in bold. The following covariates were considered: Landsat 8-based temperature (LST); Landsat 8-based NDVI (LNDVI); Landsat 8-based EVI (LEVI); Sentinel 2-based NDVI (SNDVI); Sentinel 2-based EVI (SEVI); and also the SD and kurtosis of each of those variables which are indicated by adding, respectively, SD or K as suffix to the environmental variables.

**Table 6:** Parameter estimates for the best spatial Gaussian generalized additive model explaining the mean number of *Aedes aegypti* eggs per ovitrap at Puntarenas City, Costa Rica

Parameter	Estimate	SE	*t*-value	*P-*value
Intercept	12.290	1.869	6.575	5.22E-09^a^
Kurtosis of Landsat 8-based EVI (KEVI)	2.181	0.185	11.776	2.00E-16^a^
Moranʼs I	0.031			0.193
Approximate significance of smoothed terms				

Smoothed function	Estimate *df*	Residual *df*	*F*-value	*P*-value
*s* (Landsat 8 Temperature, LST)	1.866	2.354	4.323	0.0141^a^
*s* (LST, KEVI)	0.642	0.642	65.598	4.54E-09^a^

*Abbreviation*: *df*, degrees of freedom.

a Statistically significant (*P*  <  0.05).

## 4 Discussion

Our data analysis suggests that ovitrap counts are not useful to predict *Ae. aegypti*-borne arboviral cases at the studied site. This is interesting, given the general assumption that mosquito, and more generally vector abundance measurements, are useful to understand the risk for pathogen transmission (Smith et al., 2014), and evidence about associations between vector abundance and transmission at different spatial and temporal scales for dengue (Barrera et al., 2011; Cromwell et al., 2017; Parra et al., 2018; Li et al., 2019) and other vector-borne diseases (Chaves et al., 2011, 2014a; Poh et al., 2019). Among the diverse factors that may have influenced our results we think some might be related with *Ae. aegypti* ecology, as it is related to pathogen transmission, and some might be related to the predictability of events that are rare, or that occur at different spatial scales (Levin, 1992; Levins et al., 1994). To start, one of the reasons why egg counts might not be informative about disease transmission is related to the highly likely nonlinear association between egg abundance and adult mosquito abundance. The latter stage is responsible for pathogen transmission, but adult abundance might actually decrease with egg counts because of density-dependence, especially if the relationship is similar to what has been observed between adult and larval abundance (Wilson et al., 1990; Barrera et al., 2011; Chaves et al., 2012). Similarly, the productivity of *Ae. aegypti* changes across diverse container types (Schneider et al., 2004) and how adult mosquito productivity, i.e. the number of emerging adult mosquitoes, might ultimately reflect trade-offs in oviposition habitat selection by *Ae. aegypti* (Harrington et al., 2008; Wong et al., 2012). The hypothesis of nonlinear abundance changes through the mosquito life-cycle has been supported by data fits to mathematical models looking at the abundance of *Ae. aegypti* (Chaves et al., 2012; Lana et al., 2014). More generally, ontogenetic niche shifts, such as those of mosquitoes that alternate between being aquatic and terrestrial organisms during their life-cycle, filter signals of density dependence that might be useful to predict population dynamics (Ripa & Ranta, 2007; Ranta et al., 2006; Ratikainen et al., 2008). Thus, not surprisingly, our results follow a pattern similar to what has been observed in data from other *Ae. aegypti* aquatic stages, where entomological indices based on the presence, or abundance, of either larvae or pupae, rarely correlate with dengue transmission (Bowman et al., 2014).

Mosquito eggs are not conspicuous life stages, which increases the ability to bias their counts. For example, accidently losing eggs during the manipulation of samples, might be a factor that could bias abundance estimates (Lenhart et al., 2005). The accidental loss of eggs is likely to occur when a large number of ovitraps are monitored by a mosquito surveillance crew with multiple responsibilities related to the management of vector-borne disease transmission (CENAPRECE, 2015; Tovar-Zamora et al., 2019). There is also the possibility that egg counts from ovitraps deployed over the larger area of Puntarenas could have been a better predictor for arboviral cases. However, within the Puntarenas peninsula it was clear that egg counts are highly variable at finely-grained spatial scales, and previous studies have shown this area to be the one with most persistent mosquito infestations (Marín Rodríguez & Díaz Ríos, 2012).

Nevertheless, the fact that temperature SD, a weather variable, had a significant impact on egg counts further supports that ovitraps are sensitive devices to estimate *Ae. aegypti* adult abundance, and most definitely to detect the presence of this important mosquito vector, as suggested by trials comparing multiple trap types (Resende et al., 2013; Codeço et al., 2015). For example, ovitraps had a higher sensitivity to detect *Ae. aegypti* than larval surveys (Nascimento et al., 2020). In this sense, the patterns observed in Costa Rica are similar to what has been observed for ovitraps in locations as diverse as Texas (Martin et al., 2019), Puerto Rico (Barrera et al., 2014), Brazil (Lana et al., 2014), Trinidad and Tobago (Chadee, 2009), México (Tovar-Zamora et al., 2019) and Argentina (Gimenez et al., 2020). These patterns also echo population dynamics patterns observed for adult mosquitoes, which are also sensitive to weather factors (Barrera et al., 2011; Ng et al., 2018; Scott et al., 2000). The relatively long delay of 6 weeks to see the impact of temperature and EVI might emerge *via* resonance, a phenomenon where natural populations cycles become amplified through a few generations in the presence of the right environmental conditions (Chaves et al., 2014b). For example, 6 weeks is an exact harmonic of the 3-week period of the seasonal component explaining median *Ae. aegypti* egg counts per ovitrap, the type of conditions enabling resonant increases in animal populations (Nisbet & Gurney, 1976). The best model explaining egg counts included EVI, a remotely sensed vegetation index that is associated with water in the environment (Pettorelli et al., 2005). EVI association with median egg counts was negative, probably reflecting that *Ae. aegypti* is primarily an artificial container species outside its native range in Africa (Dye, 1984; Wallace et al., 2018), needing water for the creation of its man-made larval habitats (Barrera et al., 1993; Predescu et al., 2006). However, *Ae. aegypti* larval habitats can be destroyed by an excess of rain, which can lead to a flushing of aquatic populations (Koenraadt & Harrington, 2008), ultimately reducing *Ae. aegypti* abundance (Scott et al., 2000). Still, the association is likely dynamic, as a previous, purely spatial study using high resolution satellite images for Puntarenas did not find an association between vegetation and *Ae. aegypti* abundance, which was more associated with the built urban environment (Fuller et al., 2010).

The travelling wave pattern of synchrony, where synchrony decreases with distance, and changes its sign before increasing back to its regional value (Ranta et al., 2006), to the best of our knowledge has been only reported for adults of *Armigeres subalbatus* (Coquillet), a common urban Aedini in Asia (Chaves, 2017b). This is an interesting pattern since it suggests that as much as human movement is an important factor for dengue transmission (Stoddard et al., 2009), vector movement might play a crucial role on *Ae. aegypti*-borne arboviral transmission. For example, vector control and *Ae. aegypti* dispersal ecology are prone to be asynchronous (Juarez et al., 2020). This asynchrony might ensure *Ae. aegypti* persistence in urban landscapes, as has been reported for both adult and larval *Ae albopictus* in Japan (Chaves, 2017b; Chaves et al., 2020b). Interestingly, although the pattern was not exactly the same, the synchrony of Sentinel 2-based NDVI also showed a significant decrease in synchrony at a shorter distance (170 m) than the one observed for *Ae. aegypti* eggs at 400 m. This pattern is suggestive of resonance in a two-dimensional expansion wave, a phenomenon, that to the best of our knowledge, has not been described in mosquitoes or other animal populations, but that has been described in neuron populations with hybrid synapses (Sun et al., 2013). Thus, the traveling wave pattern in *Ae. aegypti* egg count synchrony might emerge because of constrains in *Ae. aegypti* dispersal that might be conditioned, for example, by the availability of oviposition habitats (Edman et al., 1998), which in turn is linked with weather patterns (Juarez et al., 2020). This might reflect a process similar to the way neuron synapses are regulated in hybrid networks, those undergoing both chemical and electrical synapses (Sun et al., 2013). Interestingly, the evidence suggesting the emergence of this spatial pattern is further supported by the positive impact of high kurtosis, or leptokurtic, EVI on *Ae. aegypti* mean egg counts at the study site. This observation suggests that *Ae. aegypti* populations are likely to prefer less stable environments, as observed for treehole Aedini mosquitoes (Sota et al., 1994). This result also implies a nonlinear response to weather conditions, as mean egg numbers spatially increased with temperature in a fashion similar to what has been observed, temporally, for adult *Ae. aegypti* populations elsewhere (Chaves et al., 2012). For example, in Thailand, *Ae. aegypti* outbreaks, i.e. sudden changes in mosquito abundance, have been linked with the canalization of high temperatures into life history traits (Chaves et al., 2012, 2014b). More specifically, models have shown that prolonged high temperatures can reduce density-dependent mortality while simultaneously increasing fecundity and mosquito productivity (Chaves et al., 2014b).

Like mosquito egg counts, *Ae. aegypti*-borne arboviral case counts were associated with temperature SD, at multiple spatial scales. This result is in accordance with global observations for dengue, chikungunya and Zika (Wallace et al., 2018) where temperature has been shown as an important factor to explain time series of dengue cases across the globe (Stoddard et al., 2014; Chuang et al., 2017; Siraj et al., 2017; Oidtman et al., 2019). However, at the reduced spatial scale of this study we also found an impact for NDVI, a factor that has been reported as significant for dengue transmission dynamics at a country-wide scale in Costa Rica (Fuller et al., 2009) and Vietnam (Nguyen et al., 2020). This result might be related to the fact that vegetation growth is associated with several weather variables, like rainfall and temperature (Pettorelli et al., 2005). Finally, the fact that both egg and human cases were associated with temperature SD highlights the dependence of the system not only on average environmental conditions, but also on higher-order moments of environmental variability. This could emerge from Schmalhausen’s law, the biological principle stating that organisms are sensitive to both the mean and the variability of the environment (Chaves, 2017a). Overall, our results also highlight the value of environmental information derived from satellite images for the surveillance of *Ae. aegypti* and the arboviruses it transmits, as documented in Southeast Asia (Nguyen et al., 2020). This pattern has also been observed for *Culex pipiens* L. and West Nile virus transmission in the USA (Chuang et al., 2012; Chuang & Wimberly, 2012; Poh et al., 2019); and *Anopheles albimanus* (Wiedemann) abundance and malaria infection in Mesoamerica (Hurtado et al., 2018a,b; Rejmankova et al., 1993; Rigg et al., 2019). Thus, our results are particularly important in the era of big data and unlimited computational power. Nowadays, satellite data can be streamlined into algorithms for vector-borne disease transmission forecasting and dynamic risk assessments.

Finally, ovitrap-based entomological surveillance might also help to guide vector control activities in sites with spatially heterogeneous and asynchronous changes in vector abundance, like these observed for *Ae. aegypti* in the present study. Major advantages of ovitrap use for *Ae. aegypti* surveillance include their low cost and ease for systematic field deployment and monitoring (CENAPRECE, 2015; Chaverri et al., 2018; Romero et al., 2019) and the potential involvement of local residents in vector surveillance efforts (Hamer et al., 2018; Tarter et al., 2019; Sousa et al., 2020). This study was carried out during a low transmission period for dengue in Costa Rica and the rest of the Americas in 2017 and 2018 (Perez et al., 2019). This low transmission prevented the use of mosquito control during our study, because mosquito control with pesticides in Costa Rica is only used for human disease outbreak management, and based on human case numbers (Vigilancia de la Salud, 2013). However, the Costa Rican national programme for integrated vector control management is currently evaluating the use of ovitraps to guide vector control in areas prone to dengue epidemics.

## Ethical approval

This study was carried out in accordance with Article 7 from Law 9234 for biomedical research which grants the Epidemic Surveillance Division (Vigilancia de la Salud) of Costa Ricaʼs Ministry of Health (Ministerio de Salud) the ability to perform activities with the dual goal of surveillance and research, which are exempted from the approval of an Institutional Research Board, as these efforts are deemed essential for health policy planning and decision making, and do not release individually identifiable data.

## Data availability

Data are available upon reasonable request. The request needs to be accompanied by an appropriate ethical clearance in accordance with law 9234 for Biomedical Research in Costa Rica.

## Declaration of competing interests

The authors confirm that there are no known conflicts of interest associated with this publication and that there has been no significant financial support for this work that could have influenced its outcome.

## Funding

This study was funded by Costa Rica’s Ministry of Health.

## CRediT author statement

JAVC, EM, JMGA, GD, LFC and RMR designed the study. JAVC and RMR coordinated field sampling. GD, LMR, LFC, JMGA, EM, georeferenced ovitrap locations. GD and CAA verified all entomological data. LFC performed all tasks related with the remote sensing data processing and analysis. LFC made all maps. JAVC prepared Fig. 2. MRR, CAA and LMR compiled arboviral case data. LFC and GD analyzed the data. LFC, GD, MRR and LMR wrote the original draft of the manuscript. All authors read, edited and approved the final manuscript.

## Figures and Tables

**Figure:**
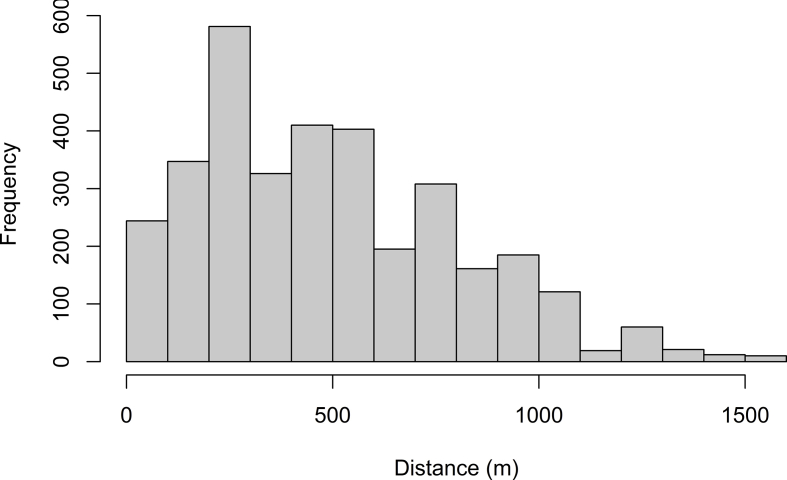
**Supplementary Figure S1.** Histogram for the distribution of the pairwise geographical distances between ovitraps.

**Figure:**
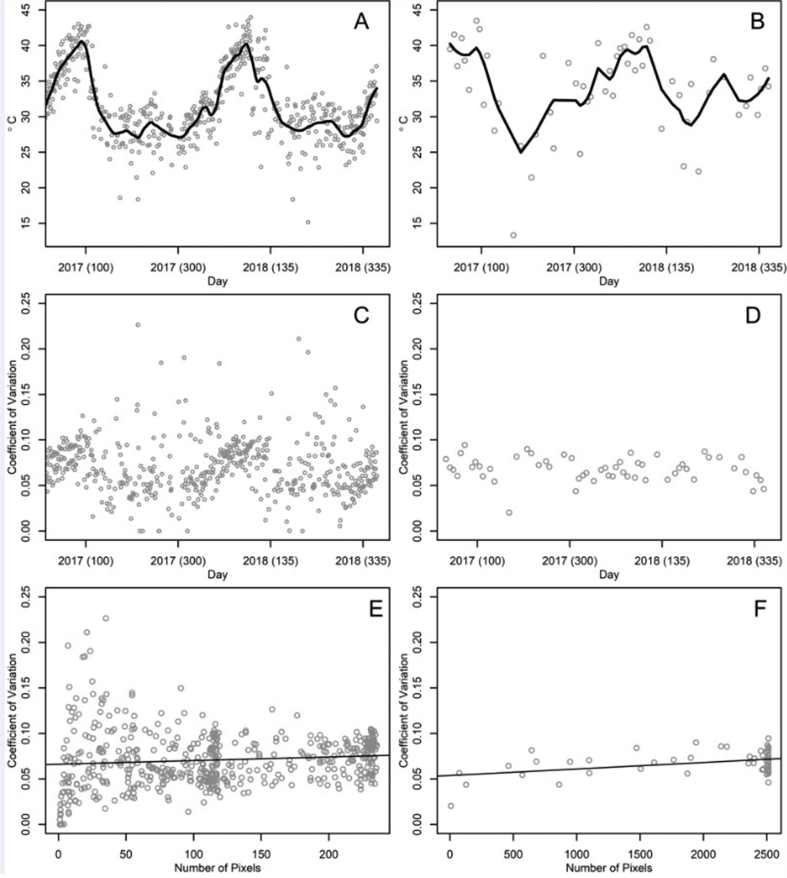
**Supplementary Figure S2.** Raw land surface temperature (LST) data. **A** MODIS LST-derived data. Each point is the average for the pixels within the Puntarenas city area for a given day and the black line is the smoothed estimate obtained through a loess regression. **B** Landsat 8 LST-derived data. Each point is the average for the pixels within the Puntarenas Peninsula area for a given day and the black line is the smoothed estimated obtained through a loess regression. **C** Coefficient of variation (CV) for MODIS LST time series during the study period. The CV ± SD was 0.0702 ± 0.0306. **D** CV for Landsat 8 LST time series during the study period. The CV ± SD was 0.0682 ± 0.0133. **E** CV for MODIS LST as a function of the number of pixels used in the estimation of each LST measurement; the line represents the bias of the estimates as a function of the number of pixels considered in LST estimation. The slope ± SE was 3.678e-05 ± 1.756e-05, which was statistically significant (*F*_(1,522)_= 4.388, *P* < 0.036). **F** CV for Landsat 8 LST as a function of the number of pixels used in the estimation of each LST measurement, the line represents the bias of the estimates as a function of the number of pixels considered in LST estimation. The slope ± SE was 7.112e-06 ± 2.146e-06, which was statistically significant (*F*(1,54) = 10.99, *P* < 0.001).

**Figure:**
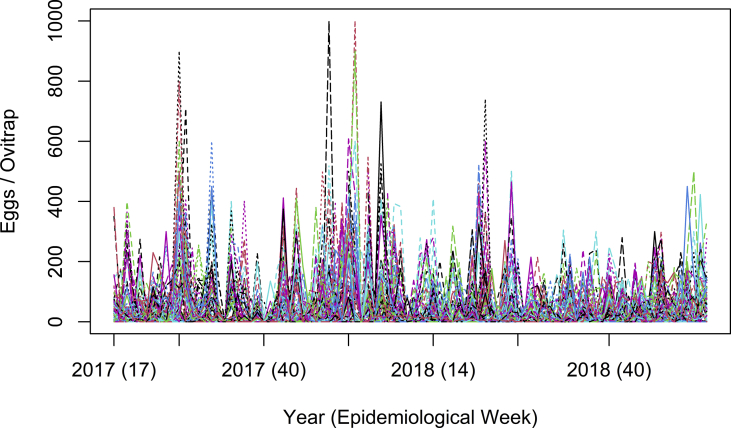
**Supplementary Figure S3.** Raw weekly egg counts for each individual ovitrap. Color and line type indicate the numbers for each individual ovitrap.

**Figure:**
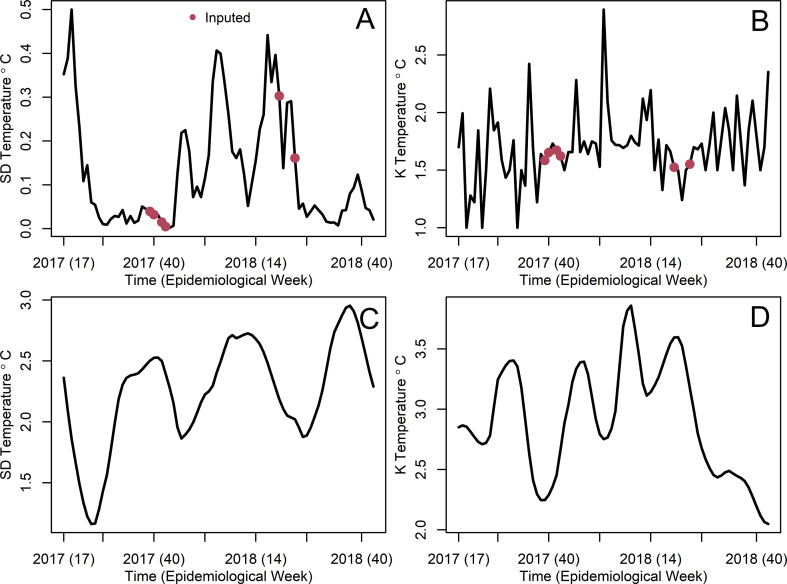
**Supplementary Figure S4.** Higher order moments of weekly land surface temperature (LST) estimates. **A** MODIS-based SD of temperature. **B** MODIS-based kurtosis (K) of temperature. **C** Landsat 8-based SD of temperature. **D** Landsat 8-based kurtosis (K) of temperature. The legend of panel **A** shows the symbol employed to indicate inputted data.

**Figure:**
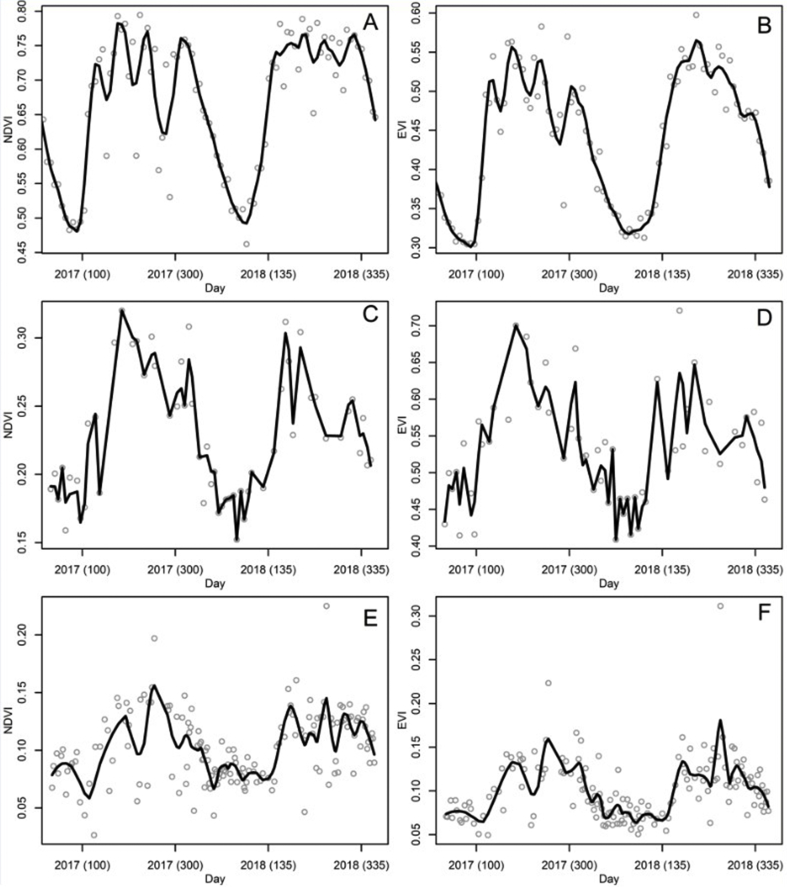
**Supplementary Figure S5.** Raw vegetation index data. **A** MODIS NDVI-derived data. **B** MODIS EVI derived data. **C** Landsat 8 NDVI-derived data. **D** Landsat 8 EVI-derived data. **E** Sentinel 2 NDVI-derived data. **F** Sentinel 2 EVI-derived data. In all panels each point is the average for the pixels within the Puntarenas Peninsula area. In all panels black lines show the smoothed estimated obtained through a loess regression. Pixel size: 250 m (MODIS images); 30 m (Landsat 8 images); 10 m (Sentinel 2 images).

**Figure:**
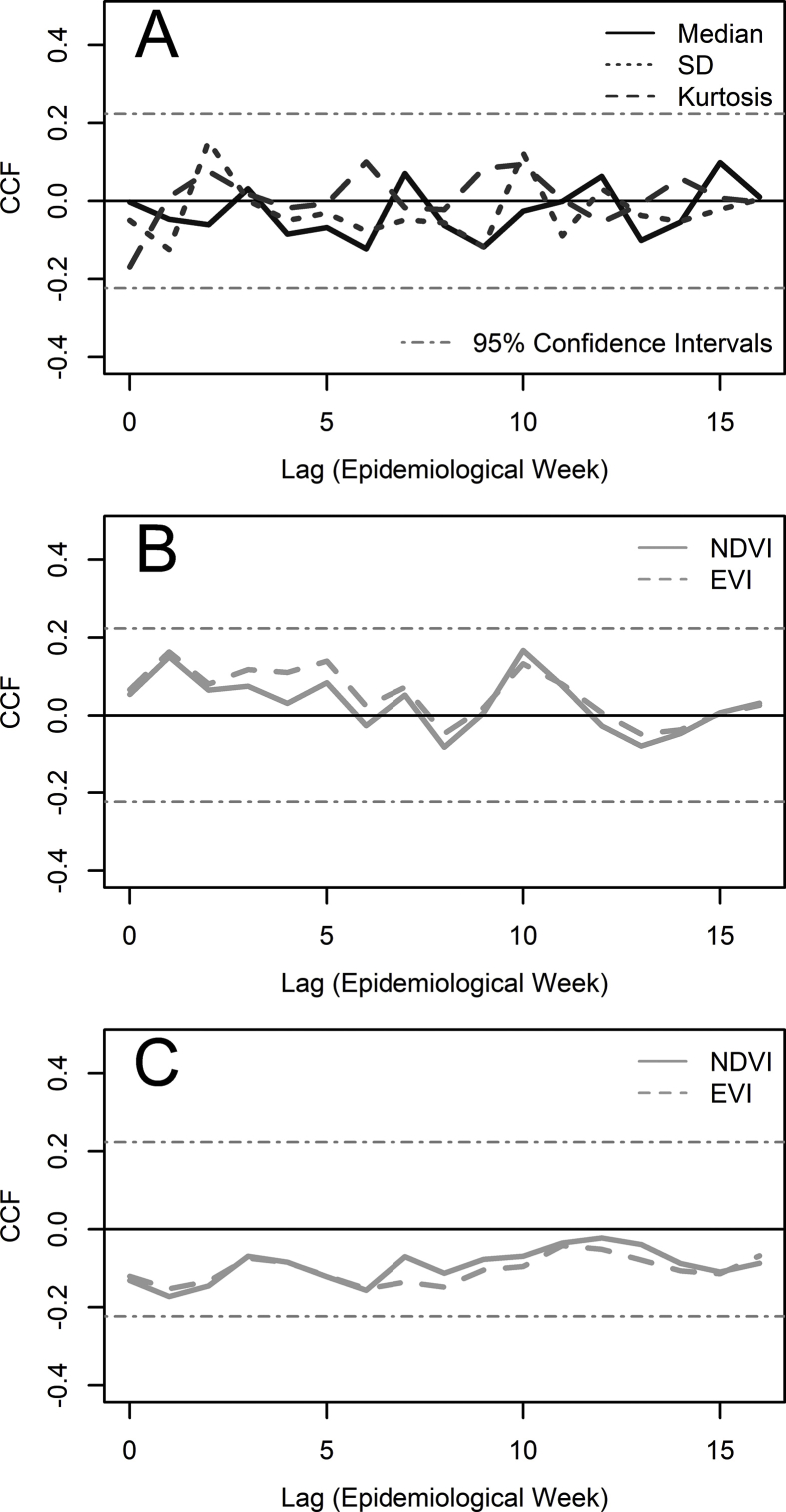
**Supplementary Figure S6.** Cross correlation functions (CCF) between **A** arboviral case number and the median, SD and kurtosis of *Aedes aegypti* egg counts, **B** arboviral case number and Sentinel 2 remotely sensed environmental variables NDVI and EVI and **C** median number of *Aedes aegypti* egg counts per ovitrap and Sentinel 2 remotely sensed environmental variables NDVI and EVI.Dot-dashed lines indicate the 95% confidence intervals outside which associations are not expected by chance. In all panels, none of the time lag correlations was significant.

**Figure:**
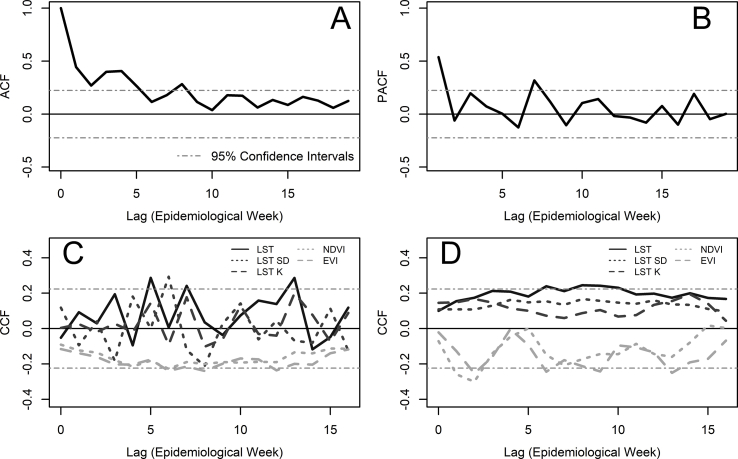
**Supplementary Figure S7.** Correlation functions. **A** Autocorrelation (ACF) and **B** Partial autocorrelation function (PACF) for the weekly mean number per ovitrap, of *Aedes aegypti* egg counts time series. Lines are identified in the inset legend of panel **B**. **C** Cross-correlation function (CCF) between weekly mean number per ovitrap, of *Aedes aegypti* egg counts and MODIS-based environmental variables, including land surface temperature, NDVI and EVI mean values, and also the SD and kurtosis of land surface temperature. **D** CCF between weekly mean number per ovitrap, of *Aedes aegypti* egg counts and Landsat 8-based environmental variables, including land surface temperature, NDVI and EVI mean values, and also the SD and kurtosis of land surface temperature. Dot-dashed lines indicate the 95% confidence intervals outside which associations are not expected by chance.
